# Genome-wide identification and expression analysis of GRAS family transcription factors in tea plant (*Camellia sinensis*)

**DOI:** 10.1038/s41598-018-22275-z

**Published:** 2018-03-02

**Authors:** Yong-Xin Wang, Zhi-Wei Liu, Zhi-Jun Wu, Hui Li, Wen-Li Wang, Xin Cui, Jing Zhuang

**Affiliations:** Tea Science Research Institute, College of Horticulture, Nanjing Agricultural University, Nanjing, 210095 China

## Abstract

GRAS proteins are important transcription factors that play multifarious roles in regulating the growth and development as well as stress responses of plants. Tea plant is an economically important leaf -type beverage crop. Information concerning GRAS family transcription factors in tea plant is insufficient. In this study, 52 *CsGRAS* genes encoding GRAS proteins were identified from tea plant genome database. Phylogenetic analysis of the identified GRAS proteins from tea plant, Arabidopsis, and rice divided these proteins into at least 13 subgroups. Conserved motif analysis revealed that the gene structure and motif compositions of the proteins were considerably conserved among the same subgroup. Functional divergence analysis indicated that the shifted evolutionary rate might act as a major evolutionary force driving subfamily-specific functional diversification. Transcriptome analysis showed that the transcriptional levels of *CsGRAS* genes under non-stress conditions varied among different tea plant cultivars. qRT-PCR analysis revealed tissue and development stage-specific expression patterns of *CsGRAS* genes in tea plant. The expression patterns of *CsGRAS* genes in response to abiotic stresses and gibberellin treatment suggested the possible multiple functions of these genes. This study provides insights into the potential functions of GRAS genes.

## Introduction

Tea is a non-alcoholic beverage accepted by an increasing number of people because of its beneficial functional components^1^. The active substances, such as tea polyphenols (especially catechins), caffeine, theanine, and polysaccharides, have been reported being a stimulant and an antioxidant, and reduce the frequency of cancers, and neurodegenerative diseases^2–4^. Tea plant (*Camellia sinensis* (L.) O. Kuntze) is an important perennial evergreen leaf-used economic crop that originated from subtropical regions^5–7^. Tea plant have a life span of over 100 years^3^.Thriving in the wild, tea plant is seriously affected in terms of growth, yield, and quality by various adverse environmental stresses, including salt, drought, cold, and heat^8–11^.

With the advent of next-generation sequencing technology, a great number of genomics, transcriptomics, proteomics and metabolomics have been reported, which provide great opportunity for the development of metabolic engineering^12–17^. Recently, the high quality and available of genome sequences of tea plant have been published, which greatly promoted the understanding the biological processes and molecular/cellular mechanisms of tea plant^18^.

Transcription factors (TFs) are critical components in physiological processes and regulatory networks of higher plants; TFs serve as a switch of gene regulation, promote or inhibit specific functional gene expression, and maintain the growth and development as well as stress responses of plants^19–21^. A large proportion of the plant genome is dedicated to TFs. For example, 7.5% of the Arabidopsis genome encodes for putative TFs^22^. A recent tea plant transcriptome analysis has indicated that at least 2543 unigenes belong to 58 TF families^23^.

The name GRAS family TFs is derived from the first three isolated members, namely, GAI, RGA, and SCR^24,25^. It was initially taken as a plant-specific TF family. However, a recent study has identified the GRAS gene family in bacteria and classified it under the Rossmann fold methyltransferase superfamily^26^. Typically, GRAS TFs share a widely conserved C-terminal GRAS domain and a variable N-terminal region, and total amino acids range from 400 to 770^24,25,27^. The highly conserved C-terminus GRAS domain is constituted by five critical conserved motifs in the following order: LHR I (leucine heptad repeat I), VHIID, LHR II (leucine heptad repeat II), PFYRE, and SAW^24,25^. VHIID along with two leucine heptad repeats (LHR I and LHR II), similar to the bZIP protein–DNA interaction, may be critical for protein–DNA or protein–protein interactions^24^. Mutations in the SAW and PFYRE regions of SLR1 and RGA proteins emerged in the dramatic phenotypic differences in Arabidopsis^28,29^. The N-terminal of the GRAS domain family is diverse and may mainly decide the specificity of corresponding proteins. As an exception, DELLA proteins contain two conserved motifs (DELLA and TVHYNP) in their N-terminal region.

Whole-genome analysis of the GRAS family revealed 33 GRAS genes in Arabidopsis^27^, 60 in rice^27^, 46 in *Prunus mume*^30^, 48 in Chinese cabbage^31^, 106 in populus^32^, 53 in tomato^33^, 48 in castor^34^, and 52 in grapevine^35^. Studies in several plants have indicated that GRAS family TFs serve diverse functions and participate in various physiological processes. For example, five widely known Arabidopsis GRAS proteins, GAI, RGA, RGL1, RGL2, and RGL3, which belong to the DELLA subfamily, negatively regulate GA signal transduction^28,36,37^. Three GRAS homologous genes, *MOC1* in rice, *Ls* in tomato, and *LAS* in Arabidopsis, participate in the formation of axillary meristem^38–40^. Two Arabidopsis GRAS genes, *AtSCR* and *AtSHR*, are mainly involved in root and shoot radial pattern formation^41–43^. Two Arabidopsis GRAS proteins, SCL21 and PAT1, positively regulate phytochrome A signal transduction, which promotes etiolated to photomorphogenic transformation^44,45^. Two *Medicago truncatula* GRAS proteins, NSP1 and NSP2, are important for efficient nodulation, which could regulate the expression of nodulation genes by forming the NSP1–NSP2 complex^46^.

Until recently, no reports have characterized GRAS family TFs in tea plant. In the present study, *CsGRAS* family genes were identified from previous tea plant genome database. Phylogenetic tree, sequence alignment, protein motif structural, and functional annotation analyses of CsGRAS TFs were performed. In addition, the expression patterns of *CsGRAS* genes among different tea plant cultivars were surveyed using available transcriptome data. Furthermore, tissue-specific expression patterns were investigated using qRT-PCR in tea plant cultivars ‘Huangjinya’ and ‘Yingshuang’, and the expression patterns in response to diverse abiotic stresses and gibberellin (GA) treatment were detected. This research provides the first evidence about tea plant GRAS TFs, which may help elucidate the molecular mechanisms underlying stress responses in tea plant.

## Results

### Identification of CsGRAS TFs in tea plant

A total of 52 CsGRAS proteins were confirmed and used for further analyses (Table S1). Hidden Markov models (HMMs) of these CsGRAS proteins were determined using the Pfam database. All of these CsGRAS proteins contain a conserved GRAS domain (PF03514.13). However, five of the CsGRAS members (CsGRAS11, -12, -16, -24 and -48) possess a DELLA domain (PF12041.7) and thus were considered DELLA proteins (Table S2).

The physical and chemical characteristics of tea plant GRAS proteins were analyzed using ExPasy (Table S3). The length and molecular weight (Mw/Da) of the deduced GRAS proteins ranged from 416 to 1340 amino acids and 46291.14 Da to 146535.66 Da, respectively. The theoretical isoelectric point (*pI*) of most CsGRASs is slightly acidic (4.88–6.84), and two GRAS proteins are alkalescent. In most CsGRAS proteins, the number of positive amino acids (Arg + Lys) is less than that of negative amino acids (Asp + Glu). The grand average of hydropathy (GRAVY) of all CsGRAS proteins varied from −0.618 to −0.074, indicating that these proteins are hydrophilic. The instability index of most CsGRAS proteins exceeded 40 (42.42–62.37), indicating that these proteins are unstable. No CsGRAS proteins were predicted to contain transmembrane helices by using the TMHHM server. Subcellular location analysis showed that most CsGRAS proteins were located in the nucleus (Table S4).

### Evolution of GRAS TFs among different plant species

**Figure 1:**
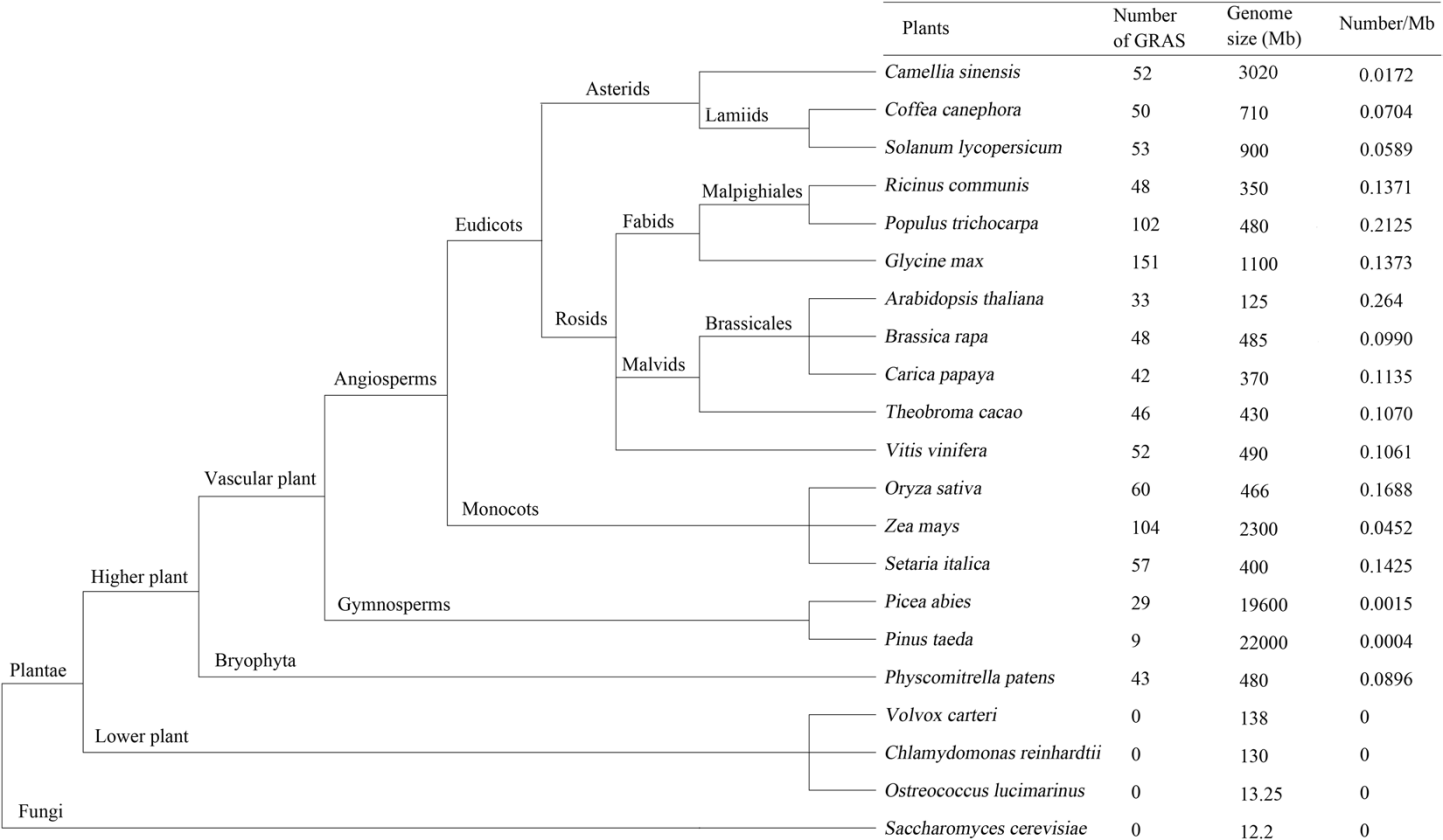
Evolution of GRAS TFs among different plant species.

A comparison analysis of GRAS TFs in different species was performed to understand the evolution of GRAS TFs among different plant species (Fig. 1). GRAS family members were only identified in higher plants; no members were found in lower plants and fungi (*Volvox carteri*, *Chlamydomonas reinhardtii*, *Ostreococcus lucimarinus* and *Saccharomyces cerevisiae*). Evolutionary analysis indicated that the GRAS proteins may have expanded since the divergence from lower plants to higher plants, and these GRAS factors are closely related to higher plant evolution. The number of GRAS TFs in tea plant (52) was similar with closely related species, *Coffea canephora* (50), *Solanum lycopersicum* (53). The number and genome size of the GRAS proteins had no evident regularity between monocots and eudicots as well as woody and herbal plants. Some species, including *Populus trichocarpa*, *Glycine max*, and *Zea mays*, exhibited a relatively large number of GRAS family members (i.e., over 100). The density of the GRAS proteins to genome size was rather low in several plant species, which are particularly evident in two gymnosperms, namely, *Picea abies* (0.0015 number/Mb) and *Pinus taeda* (0.0004 number/Mb). This occurrence is mainly due to the large genome size of these two species.

### Phylogenetic analysis of GRAS proteins in tea plant, Arabidopsis, and rice

A phylogenetic analysis of GRAS proteins from tea plant, Arabidopsis, and rice was conducted to clarify the evolutionary relationships among tea plant GRAS proteins. We acquired 34 GRAS proteins in Arabidopsis and 60 in rice, which had been proven in a previous study^32^. Meanwhile, some members possess atypical GRAS domains, and some of them are severely truncated. For example, the domain sequences of some GRAS proteins are less than 100 amino acids (e.g., ΨOsGRAS2, ΨOsGRAS3, ΨOsGRAS5, ΨOsGRAS7), whereas the approximate number of typical GRAS domain is 350 amino acids. The short or atypical sequences may be putative pseudogenes and derived from ancient pseudogenization events^32^. Considering that incorporating these fragments results in low reliability in multiple sequence alignment and phylogenetic analysis, we excluded these fragments for later analyses. Finally, 33 GRAS members in Arabidopsis and 50 in rice were identified (Table S5). Similar to those in tea plant, these proteins possess a typical GRAS domain.

**Figure 2:**
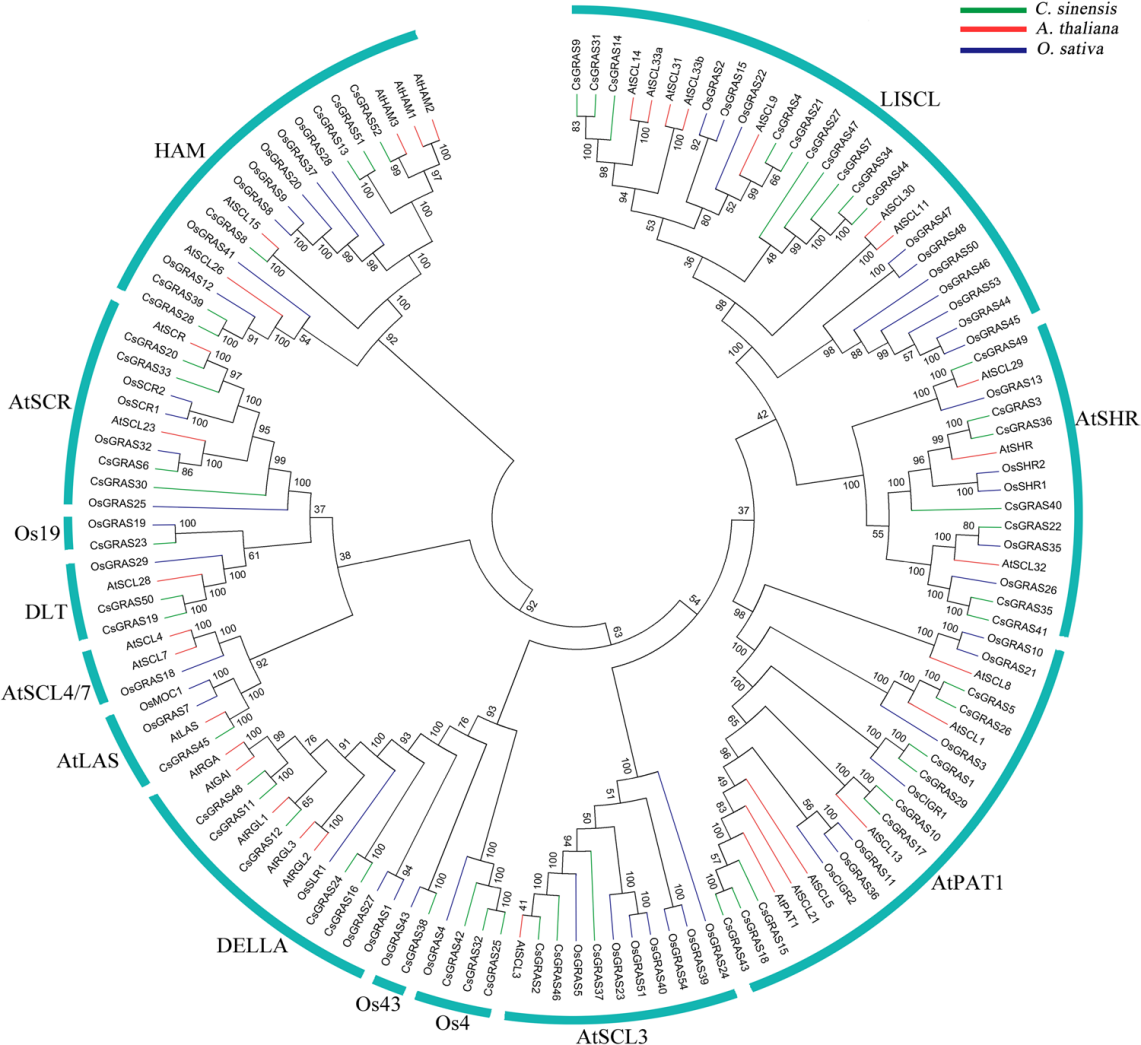
Phylogenetic analysis of GRAS proteins from tea plant, Arabidopsis, and rice. Full-length GRAS protein sequences were aligned using Clustal 1.83, and the phylogenetic tree was constructed using MEGA 5.05 by the neighbor-joining (NJ) method with 1000 bootstrap replicates.

Phylogenetic trees were constructed with alignments of 135 full-length GRAS proteins, including 52 from tea plant, 33 from Arabidopsis, and 50 from rice, and the neighbor-joining (NJ), minimum evolution (ME), and maximum likelihood (ML) algorithms were employed (Fig. 2 and Figs S1 and S2). The NJ and ME trees exhibited similar topologies. Compare with the NJ and ME trees, the ML tree exhibited only minor modifications. In this study, the NJ phylogenetic tree was used for further analysis. On the basis of the phylogenetic analysis and existing research, all GRAS proteins were grouped into 13 subfamilies, namely, HAM, DELLA, AtSCL3, DLT, AtSCR, AtLAS, AtSCL4/7, AtSHR, AtPAT1, Os4, Os19, Os43, and LISCL. The CsGRAS proteins were unevenly distributed. For instance, most of the CsGRAS proteins were distributed in the AtPAT1 (9 members), and LISCL (10 members) subfamilies, whereas no members were found in the AtSCL4/7 subfamily.

### Estimation of functional divergence

To investigate the level of functional divergence between gene clusters of the GRAS subfamily, type I and type II functional divergence were estimated using DIVERGE (v2.0) software on the basis of the ML algorithm^47–49^, which provides a sequence-based method to identify cluster-specific amino acid sites that are likely responsible for functional changes after gene duplication. Type I functional divergence between two duplicate clusters indicates that some amino acid residues are highly conserved in one duplicate cluster but highly variable in another cluster, suggesting that these sites may have experienced shifted functional constraints^47^. Type II functional divergence between two duplicate clusters indicates that some amino acid residues are highly conserved in both duplicate clusters but exhibits distinct biochemical properties, suggesting that these sites may be responsible for functional specification in different subfamilies^50^. On the basis of the GRAS protein NJ tree, GRAS subfamilies HAM, DELLA, AtSCL3, AtSCR, AtSHR, AtLAS, DLT, AtPAT1, Os4, and LISCL were used in the present study as input for the DIVERGE2 analysis, whereas Os19, Os43, and AtSCL4/7 were excluded because of their small size (Table 1).

**Table 1:** Analysis of functional divergence between subfamilies of GRAS proteins among tea plant, Arabidopsis, and rice.

	Type-I				Type-II		
	θ_I_	SE	LRT	Q_k_ > 0.95	θ_II_	SE	Q_k_ > 0.95
AtPAT1/AtSHR	0.7	0.069923	100.220992	16	−0.189746	0.47519	0
AtPAT1/HAM	0.7544	0.064688	136.00456	24	0.316559	0.390334	46
AtPAT1/AtLAS	0.6144	0.102491	35.935966	3	0.136477	0.278435	52
AtPAT1/DLT	0.628	0.120907	26.978521	1	−0.11259	0.293901	4
AtPAT1/AtSCR	0.4576	0.063629	51.719732	5	−0.031956	0.272601	10
AtPAT1/DELLA	0.5576	0.06274	78.988284	8	0.262206	0.224824	45
AtPAT1/Os4	0.2584	0.130515	3.919778	0	−0.094026	0.257403	1
AtPAT1/AtSCL3	0.3128	0.08113	14.865175	0	−0.320102	0.453195	0
AtPAT1/LISCL	0.5328	0.06378	69.784981	7	0.104625	0.393208	42
AtSHR/HAM	0.3072	0.097946	9.837073	0	0.58637	0.41215	94
AtSHR/AtLAS	0.1744	0.16799	1.077776	0	−0.568076	0.538326	0
AtSHR/ DLT	0.6984	0.179402	15.154855	2	−0.186096	0.436951	0
AtSHR/AtSCR	0.5488	0.117272	21.899845	0	−0.119911	0.384469	0
AtSHR/DELLA	0.4408	0.091315	23.302277	1	−0.18941	0.362443	0
AtSHR/Os4	0.692	0.180505	14.69719	2	−0.143704	0.368777	0
AtSHR/AtSCL3	0.564	0.094934	35.294819	2	−0.435739	0.630512	0
AtSHR/LISCL	0.7848	0.064258	149.16303	24	0.776996	0.222206	0
HAM/AtLAS	0.1728	0.133267	1.68129	0	−0.66935	0.614979	0
HAM/DLT	0.4512	0.184097	6.006845	0	−0.331148	0.531666	0
HAM/AtSCR	0.5088	0.105425	23.291903	0	−0.034738	0.413279	3
HAM/DELLA	0.5776	0.086033	45.073668	3	0.301834	0.306603	54
HAM/Os4	0.8872	0.198931	19.890097	6	−0.041471	0.415968	14
HAM/AtSCL3	0.7728	0.094339	67.104971	11	0.308897	0.48518	45
HAM/LISCL	0.7688	0.065637	137.193874	26	−0.331015	0.655931	0
AtLAS/DLT	0.001	0.022361	0	0	−0.526661	0.276731	0
AtLAS/AtSCR	−0.281576	−6.296225		2	−0.617643	0.273432	0
AtLAS/DELLA	0.2696	0.117444	5.269648	0	−0.301686	0.220025	0
AtLAS/Os4	0.632	0.259499	5.931472	0	−0.040479	0.193327	18
AtLAS/AtSCL3	0.468	0.171671	7.431882	0	−0.526521	0.441812	0
AtLAS/LISCL	0.7904	0.112978	48.944725	8	−0.164104	0.399641	5
DLT/AtSCR	0.2864	0.140427	4.159547	0	−0.209416	0.191666	0
DLT/DELLA	0.6456	0.124421	26.923808	3	−0.142312	0.188879	0
DLT/Os4	0.7496	0.262913	8.128988	0	0.186205	0.137556	44
DLT/AtSCL3	0.7584	0.19015	15.907627	1	−0.341788	0.388346	0
DLT/LISCL	0.7376	0.121078	37.111799	5	−0.297637	0.395422	0
AtSCR/DELLA	0.3264	0.083722	15.199391	1	−0.263457	0.204471	0
AtSCR/Os4	0.2024	0.106418	3.617345	0	−0.117367	0.154718	6
AtSCR/AtSCL3	0.464	0.09441	24.15461	1	−0.198747	0.330886	0
AtSCR/LISCL	0.5304	0.073653	51.859123	4	0.042399	0.359332	2
DELLA/Os4	0.524	0.115246	20.673204	2	−0.241542	0.151758	25
DELLA/AtSCL3	0.6624	0.093931	49.730757	4	−0.174281	0.310198	0
DELLA/LISCL	0.8856	0.07198	151.373785	44	0.192837	0.277102	56
Os4/AtSCL3	0.344	0.210423	2.672572	0	−0.395914	0.340568	0
Os4/LISCL	0.1272	0.147236	0.746357	0	−0.390697	0.367366	0
AtSCL3/LISCL	0.4712	0.067431	48.830625	6	−0.319227	0.526905	0

**Figure 3:**
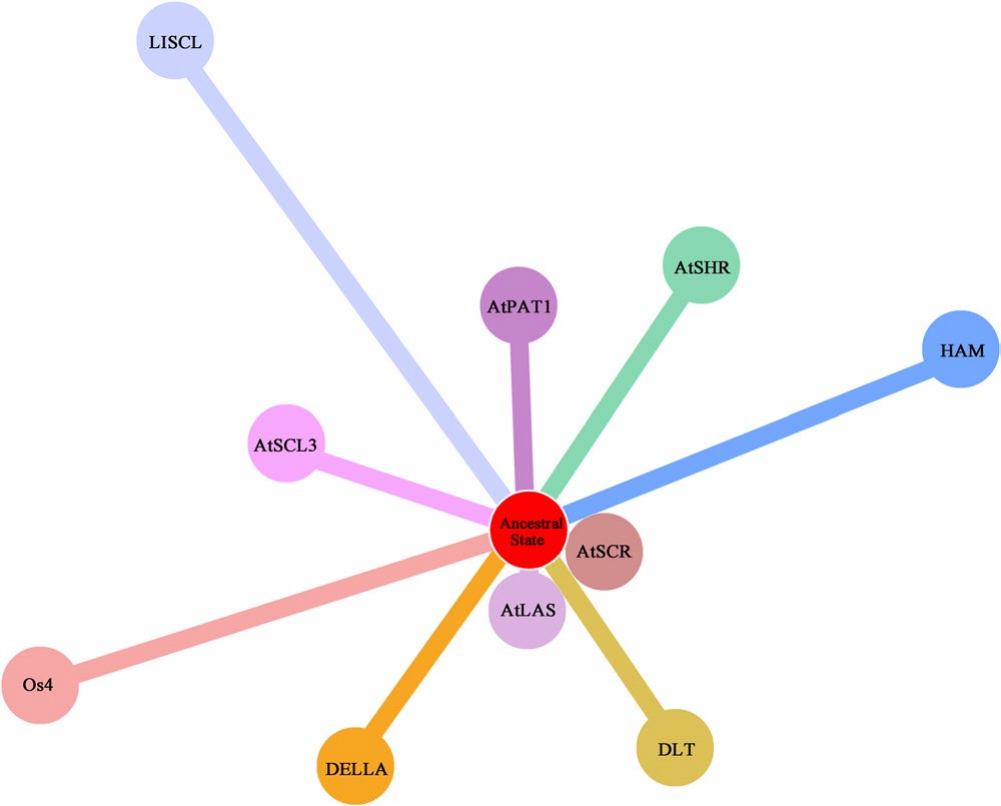
Tree-like topology of GRAS proteins of each subfamily in terms of the functional distance *b*_F_.

Functional distance analysis was conducted and a star-like tree in terms of the functional branch length (b_F_) of each subfamily was generated (Fig. 3) to examine the pattern of type I functional divergence for each pair of subfamilies. The functional branch lengths of the subfamilies were measured and ranked as follows: b_F_ (LISCL, 1.01) > b_F_ (Os4, 0.83) > b_F_ (HAM, 0.78) > b_F_ (AtSHR, 0.52) > b_F_ (DELLA, 0.48) > b_F_ (DLT, 0.44) > b_F_ (AtSCL3, 0.43) > b_F_ (AtPAT1, 0.35) > b_F_ (AtLAS, 0.06) > b_F_ (AtSCR, 0.01). LISCL exhibited the longest functional branch, indicating that the evolutionary conservation may have been changed at many sites for this subfamily and that the derived functional state may be far away from the ancestral state. By contrast, the bF for AtSCR was virtually zero, suggesting that the evolutionary rate of each site has not changed substantially since duplication and that this subfamily is likely to have inherited the ancestral function.

The coefficients of type I functional divergence (θ_I_) between any two subfamilies were significantly greater than 0 (*p* < 0.05, Table 1), suggesting that site-specific rate shift after gene duplication is a common phenomenon in the evolution of the GRAS family. The coefficient of type II functional divergence (θ_II_) was smaller than 0 between most pairs but showed relatively high standard errors. To further explore the pattern of functional divergence between GRAS subgroups, posterior analysis was conducted to identify the critical amino acid sites (CAASs) that may be highly relevant to functional divergence. To reduce possible false positives, CAASs between gene clusters were identified using posterior probability (Q_k_) > 0.95 as the cut-off. Results showed that the numbers of CAASs were considerably varied among each comparison (Table 1). Some CAASs were supposed to be responsible for type I functional divergence, of which 16, 24, 24, 11, 26, and 44 CAASs were identified for pairs AtPAT1/AtSHR, AtPAT1/HAM, AtSHR/LISCL, HAM/AtSCL3, HAM/LISCL, and DELLA/LISCL, respectively. In contrast to type I functional divergence, 52, 94, 54, and 56 type II-related CAASs were identified for pairs AtPAT1 /AtLAS, AtSHR/HAM, HAM/DELLA, and DELLA/LISCL, respectively. No CAASs were identified for type I functional divergence between AtSHR/HAM and DLT/Os4, whereas 94 and 44 respectively predicted sites existed for type II functional divergence. Thus, the rapid change in amino acid physiochemical properties can be mainly attributed to the evolutionary force driving the functional divergence between this pair and secondarily to the shift in evolution rate. This research indicated that site-specific shifts in evolutionary rate and changes in amino acid property are inconsistent acts on the GRAS subfamily over the course of evolution.

### Analysis of the sequence alignment and conserved motifs of the GRAS proteins

Multiple alignments indicated that all GRAS proteins contain a widely and conserved C-terminal GRAS domain, which could be divided into five parts (LHRI, VHIID, LHRII, PFYRE, and SAW) (Fig. S3), and a highly variable N-terminal domain (data not shown) as described in previous studies^24,25,27^. The five conserved motifs could be further subdivided into small distinct units. For example, both LHRI and LHRII are composed of two repeat units (A and B); VHIID can be divided into three units (A, B, and C); PFYRE contains three distinct parts (P, FY, and RE); SAW is composed of four units (RVER, W-G, L-W, and SAW). Overall, the results are slightly similar to previous statements.

**Figure 4:**
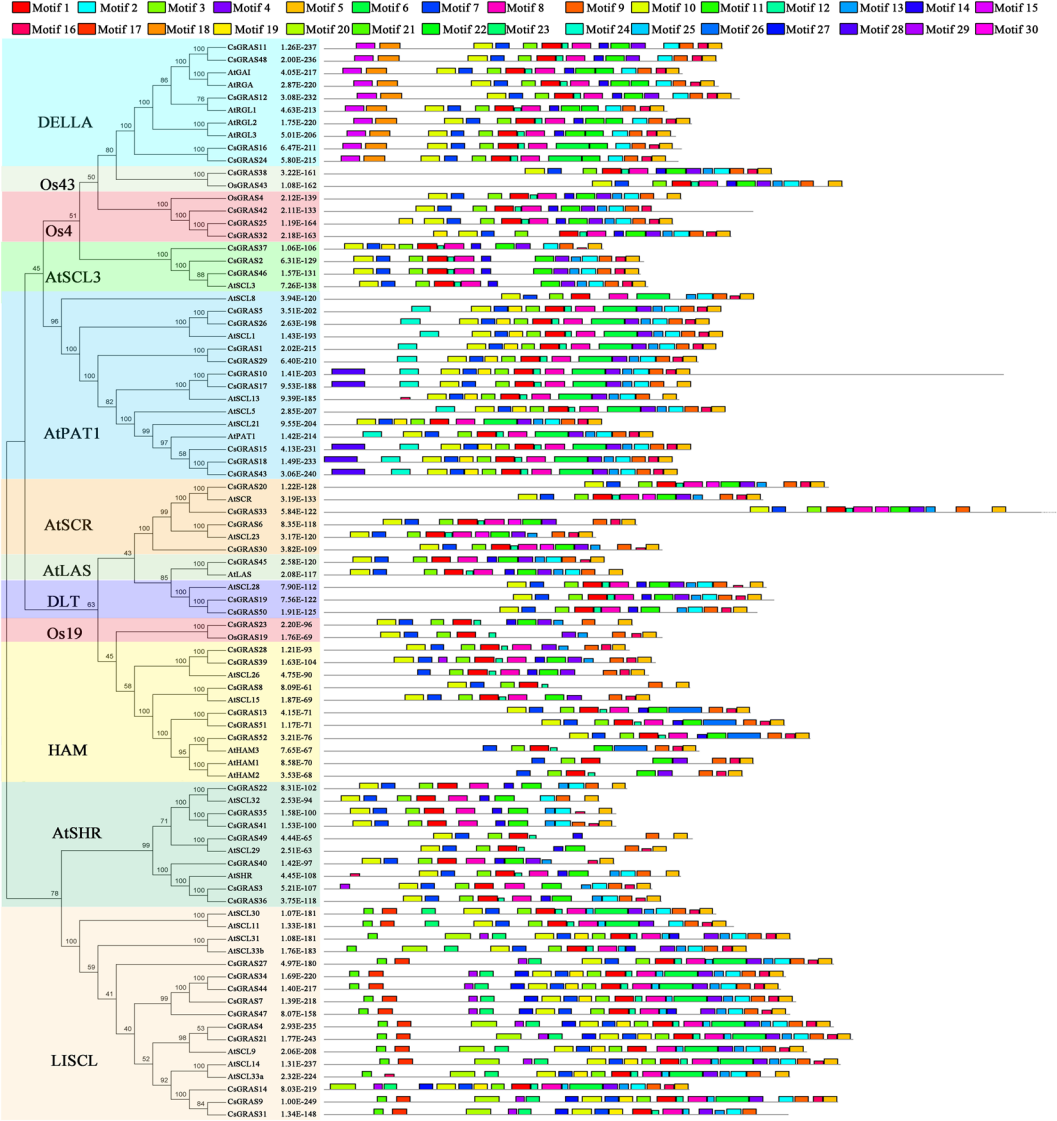
Motif analysis of CsGRAS proteins from tea plant and Arabidopsis. A phylogenetic tree was constructed by MEGA5.0. Different subfamilies are marked with different color backgrounds. Motifs in the GRAS proteins were elucidated by MEME. Different motifs are represented by different colored numbered boxes.

To further examine the sequence features of GRAS TFs from tea plant, a comparative analysis of the conserved motifs was performed between tea plant and Arabidopsis GRAS proteins. Thirty motifs were predicted to reveal the details of the GRAS protein structure using the MEME program (Table 2 and Fig. S4). In general, GRAS proteins clustered in the same subgroups share similar motif compositions (Fig. 4), indicating functional similarities among members of the same subgroup. Nearly all of the GRAS members contained motifs 1, 3, 5, 7, 9, and 10, suggesting the important roles of these motifs in the GRAS gene family. Motifs 17, 20, 21, 23, 25, and 27 were only found in subgroup LISCL; 26 was only found in subgroup HAM; 2 was completely lost in subgroups AtSCR, Os19, and HAM; 15, 18, 22 were only found in subgroup DELLA; 24 and 28 were only found in subgroup AtPAT1; 30 was only found in subgroup AtSCR; 6 was found and 11 was completely lost in subgroups AtPAT1 and LISCL. The differences in motif distribution among the subgroups of GRAS genes revealed that the functions of these genes may have diverged during evolution.

**Table 2:** Conserved motifs identified from tea plant and Arabidopsis GRAS proteins by MEME software.

Motif	E value	Width	Sites	Amino acid sequence composition of motif
1	5.8e-1303	86	29	[VLI]H[IV][IV]D[FL][DG]IX[QY]GFQWP[TSA]L[IM]QAL[AS]ARPGGPP
2	1.8e-792	64	23	C[LF][GA]R[ED][IV][VM]N[VI][VI]ACEGA[ED]RVER[HP]ET
3	1.5e-770	85	21	[FL]YEV[CS]P[YF]LKF[GAS][HY]FTANQAILE
4	8.4e-617	63	22	[HTY]N[ATG][PA]FF[LV][TG]RFREAL[HF][YH]YS[AS][LMI]F[DE]
5	9.8e-718	84	21	DDG[CA]L[LV]LGW[KQ][GD]RPL[VI][AT]ASAW[KR]
6	4.8e-709	30	50	P[GD]E[AV]L[AV]VN[FCS][LAP][FY][RQ]L[HR][HN][ML][PL]DE[ST]VS[VT]E[NS][PH]RD[RI][LV]L[RNK][LM][VI][KR][SRK][LI][NS]P[KD][VLI][VF][TI][LH][VG][EI]Q[EN][GS]N
7	1.9e-642	86	21	PXGDPMQR[LV]AAYFAE[AG]LAARL
8	7.3e-704	78	29	[ED]G[LV]EE[TV]GRRLAK[FLY]AES[LF][GN]VPFE[FY][HN][AG][VI]AXK
9	2.0e-503	84	21	X[KQ]WRXR[MFL]XRAGF[RK]PVPLSSXA
10	2.8e-599	82	29	DL[VR][QH]LL[LI]ACA[EQ]A[VI][AS]X[GN][DN]LXLAXXLLK[EQ][LI]R
11	1.40E-304	48	29	LX[SR]X[GS][SA][LI]XS[FV]L[RS]X[ILV][KR]SL[SN]P[KR][IV]V[TV][VL]VE[QE][ED][AS]
12	2.80E-209	75	11	H[LV]R[IL]TG[IV][GD]X[PS]Q
13	1.90E-207	60	15	[DE][AT]TLPR[ED][SD]KER[IL][NK]VE
14	1.50E-158	41	15	[RKS]E[GD]EA[LV][AV][VI]N[SC]V[FL]QLH
15	6.00E-127	10	29	D[EG]LLA[VG][LA]GYKV[RK][SA]S[DE][ML]A[DE]VAQ[KR]LE[QR]LE[MET][VM]
16	1.20E-120	75	15	KL[LV][LK]XXYXDG[YF]R[VLI][ED]E
17	1.50E-99	13	22	ALQA[AT]E[KR]S[FL]Y[DE][VA][LI]G[EK][KQ]YPPS[PS][SN]
18	3.20E-118	10	32	[DA][GD][LI]S[HNQ]L[AL][NT][DE][TA]VHYNPS[DE]L[SY][SG]W[VL][DEQ]S[ML][LI][ST][ED]LN[PNQ][PL]
19	1.30E-117	27	21	G[ST]G[ST]Q[IL]YKALK[CS]K[ER]P[SA][AS]A[ED]LL
20	5.10E-104	10	38	V[SK][NDS][MI]F[SN][DN][SA][EQD][SL][VAI][LW]QF[KR][RK]G[VM]EEA[SN][KR]FLP[KN][SN][NDS]Q[LW][IFV]I[DN][LF][DEG][SV][YEN]
21	2.10E-74	16	15	SDAVLKYI[NS]Q[MI]LMEE
22	9.90E-76	7	27	HN[GQ]P[VE]FLDRF[TN]E[AS]L[HY]YYS[TS][LM]FDSLE[GD][CV]
23	5.20E-65	17	21	DLEE[EG]RS[NS]KQ[SLP]A[VS][YF][VT][ED][ED]S[DE][LE][SQ]
24	6.80E-53	12	29	SGSC[VI]TDD[GE][ND]E[LFM]R[HLY]K[LI][QR]ELE[TR][AV][LM][LM]G[PD][DE][SD]D
25	7.50E-44	17	11	W[ED]TIK[LI][ED][DE]LK[IL]
26	1.10E-41	4	50	R[AT]DLP[FI][SP][HNQ][HQ][LV][LAI][HL][AS][IL][QH][SY][YH][EST][IAN]L[LF][DE]S[IL]DAVN[AV]N[LS][DE][AT][ML][NQ]KIE[KR]FL[VIL]QP[REG]IE[KS][IL]V
27	2.80E-38	9	21	QQNG[QP][PS][KH]G[SP][SN]G[GAR]K[GN]R[GA][KR]KQ[GN]N
28	5.60E-44	5	50	[GMS][SFI][HN][GART][LF]Y[YH][QE][PT][MGK][QLP][EQD][VI]E[PADS]Y[CPS][FILWY][PS][PQS][FCS]Q[IANT][LF][DEHT][HNQ][NQG][LSV][CFR][SYP][DSE][ND][GSD][SK][QH][GA][TS][HQ][VFS][SL][FIV][QH][TS][SCY][NDGL][EQ][KPL][YGH][CFT][TV]
29	3.50E-27	14	14	[PS]N[GI]S[RK][GE][KR]K[NHT]HHR[ED][DEN]
30	1.00E-26	6	21	DP[ES][RQ]LNVRK[GR]EAV[AV][VI]HW[LM]QHS

### Functional identification and interaction network of the GRAS proteins in tea plant

Annotations of the CsGRAS factors were retrieved from tea plant transcriptome databases and were predicted from integrated function annotation databases (COG, GO, KEGG, Swissprot, TrEMBL, Nr, and Nt) (Table S6). The predicted *CsGRAS* genes possibly participate in diverse development processes, such as “floral organ morphogenesis”, “regulation of seed dormancy process”, “post-embryonic development”, “seed germination”, “post-embryonic morphogenesis”, “reproductive structure development”, “regulation of post-embryonic development”, “organ development”, “petal formation”, “sepal formation”, “radial pattern formation” and “leaf development”. *CsGRAS* genes may be involved in various abiotic and biotic stresses, such as, “response to xenobiotic stimulus”, “hyperosmotic response”, “response to salt stress”, “response to temperature stimulus”, “response to osmotic stress”, “response to red or far red light” and “defense response to bacterium”. As transcriptional regulators, *CsGRAS* genes may be associated with the biosynthetic and signaling pathways of multi-hormones, such as GA, jasmonic acid, abscisic acid, salicylic acid, auxin, and ethylene.

**Figure 5:**
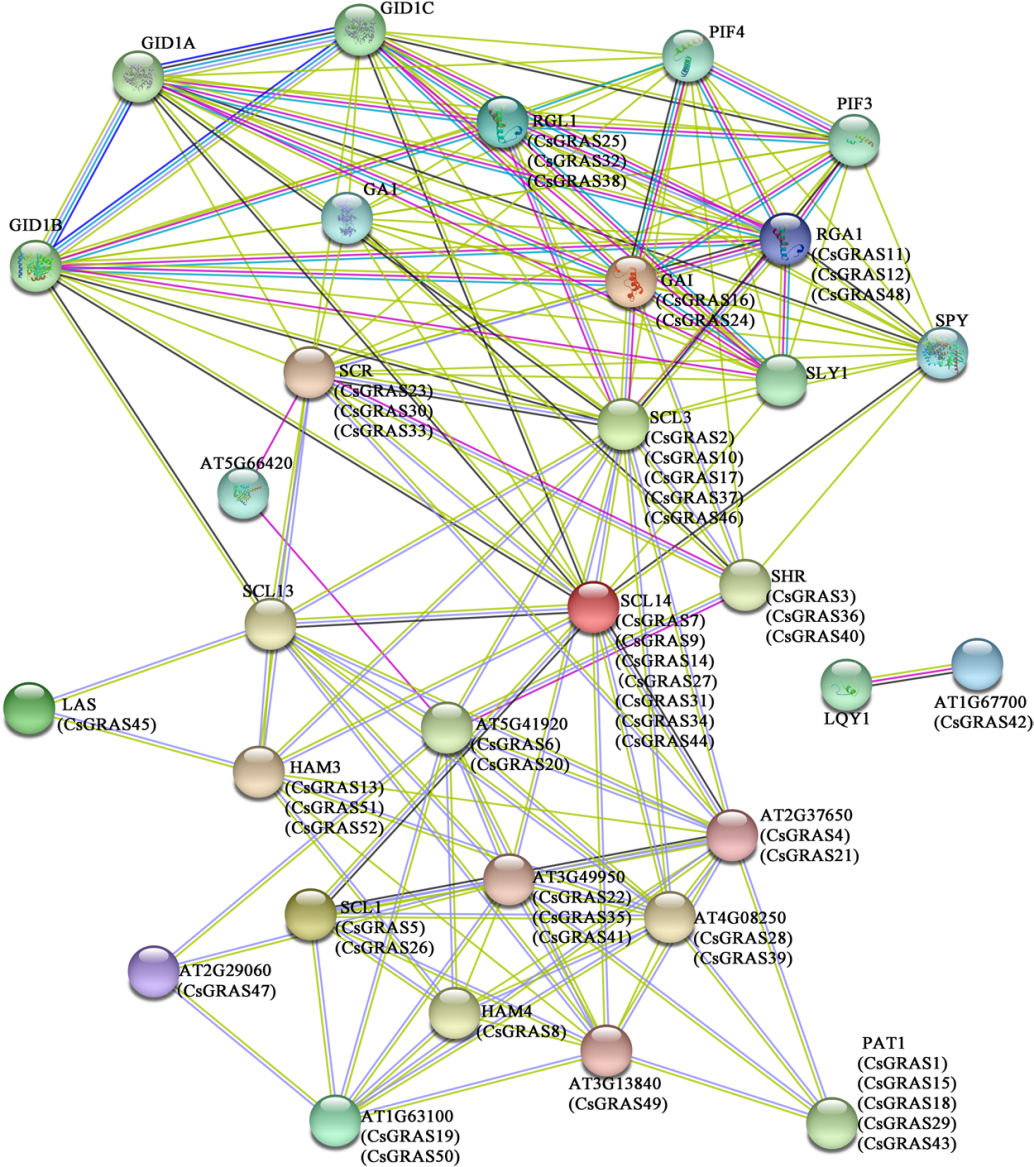
Functional interaction networks of CsGRAS proteins in tea plant according to orthologs in Arabidopsis.

To understand further the interactions of the GRAS genes in tea plant, an interaction network was built using STRING software on the basis of the orthologs in Arabidopsis (Fig. 5). The homologous proteins with the highest bit score were considered STRING proteins. SCL13 acts as a positive regulator of continuous red light signals downstream of phytochrome B (PHYB) and is required for the regulation of hypocotyl elongation during de-etiolation. SCL13 may also be required to modulate phytochrome A (PHYA) signal transduction in a phyB-independent manner. PAT1 (CsGRAS1, 15, 18, 29, and 43) may be involved in PHYA signal transduction. SHR (CsGRAS3, 36, and 40) is involved in radial pattern formation in roots and is required for normal shoot gravitropism, which could directly control the transcription of SCR(CsGRAS23, 30, and 33) and of MGP, RLK, TRI, NUC, and SCL3 (CsGRAS2, 10, 17, 37, and 46) when associated with SCR. GID1A, GID1B, and GID1C interact with specific DELLA proteins that are required for GA signaling that controls root growth, seed germination, and stem elongation. Compared with other DELLA proteins, RGA1 (CsGRAS11, 12 and 48) is the most sensitive to GA application, whereas GAI (CsGRAS16 and CsGRAS24) is less sensitive to GA^51^.

### Expression profiles of *CsGRAS* genes in four tea plant cultivars

**Figure 6:**
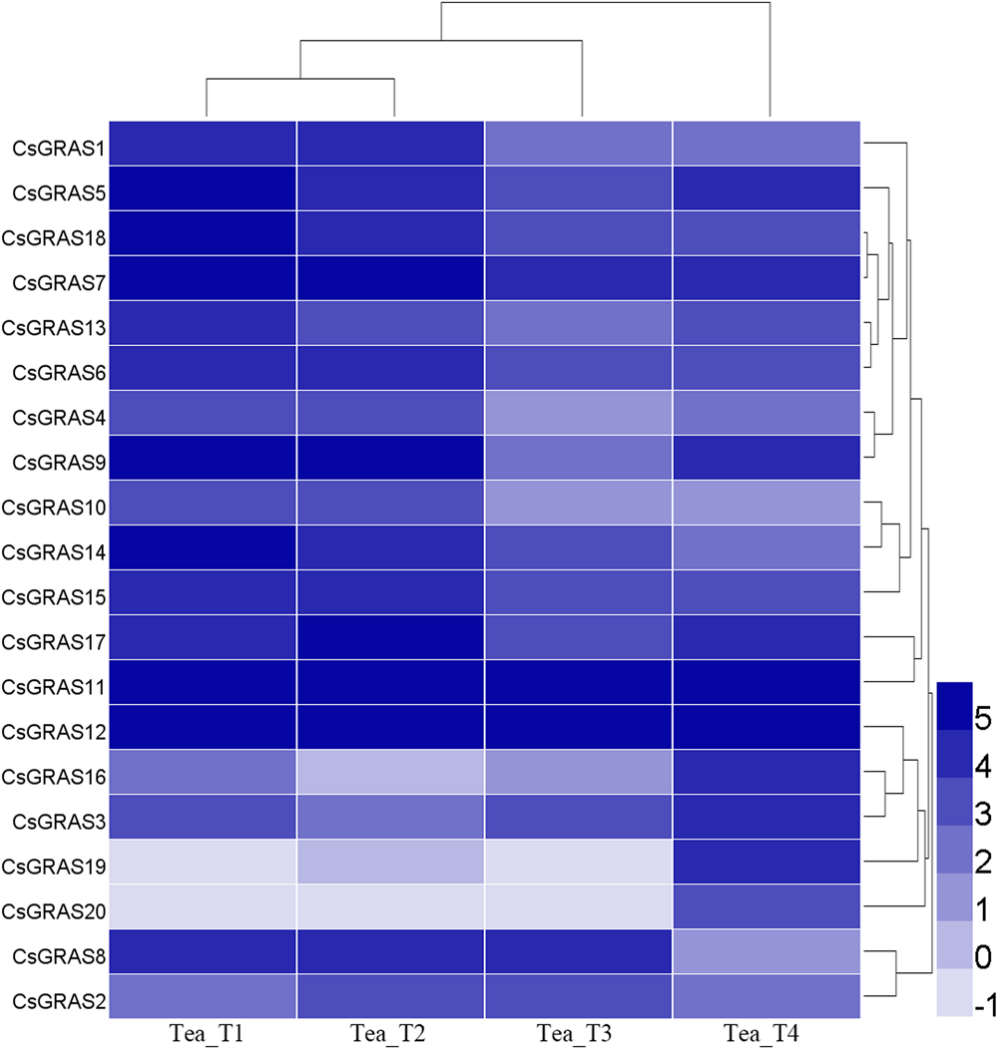
Heatmap representation for the expression of *CsGRAS* genes among four tea cultivars. RPKM values were identified from RNA-seq data and normalized by log2 transform. The color scale represents log2 transformed values. Light blue represents low expression. Dark blue represents high expression.

Using RNA sequence transcriptome data, we investigated the transcript levels of the putative GRAS genes among different tea plant cultivars under normal condition. A heat map was exhibited to assess the transcription profile of the *CsGRAS* genes among four tea plant cultivars on the basis of the reads per kilobase per million (RPKM) values (Fig. 6 and Table S7). All of the 20 *CsGRAS* genes were widely expressed across a variety of cultivars, apart from *CsGRAS20*, which was not detected in Tea_T3. This gene may have lost or weakened its functions in Tea_T3 during evolution that its expression became either too low for detection or had spatial and temporal patterns. Overall, most *CsGRAS* genes (except for *CsGRAS19* and *CsGRAS20*) exhibited a relatively high level (RPKM > 1) in all of the four tea plant cultivars. Some genes, such as *CsGRAS7*, *11*, and *12*, were stable and highly expressed. Some obviously exhibited cultivar specificity. For example, *CsGRAS3*, *16*, *19*, and *20* displayed higher expressions in Tea_T4 than in the three other cultivars; on the contrary, *CsGRAS2* and *CsGRAS8* showed a lower expression in Tea_T4 than in the others. In addition, *CsGRAS1*,* 4*,* 6*, *7*, *9*,* 10*, *13*, *14*, *15*, and *18*, displayed higher expression levels in Tea_T1 and Tea_T2 than in Tea_T3 and Tea_T4. Among the three genes encoding DELLA proteins, *CsGRAS11* and *CsGRAS12* were stable and highly expressed in all tea plant cultivars, whereas *CsGRAS16* was variable. The diversity of expression indicates that the *CsGRAS* genes may show functional differentiation among different tea plant cultivars.

### Expression profiles of *CsGRAS* genes in different tissues of tea plant

The expression patterns of *CsGRAS* genes in different tissues or different development stages (root, stem, bud, first leaf, second leaf, and third leaf) of two tea plant cultivars (‘Huangjinya’ and ‘Yingshuang’) were analyzed using qRT-PCR to explore the potential functions of *GRAS* genes during the vegetative development of tea plant. Eighteen *CsGRAS* genes were investigated. The 18 genes were representative and can explain the expression profiles of genes from different subgroups. All of the desigened primers were suitable for qRT-PCR based on the corrected sizes of PCR amplified band and the validated by PCR paired-end sequencing (Figs S5 and S6).

**Figure 7:**
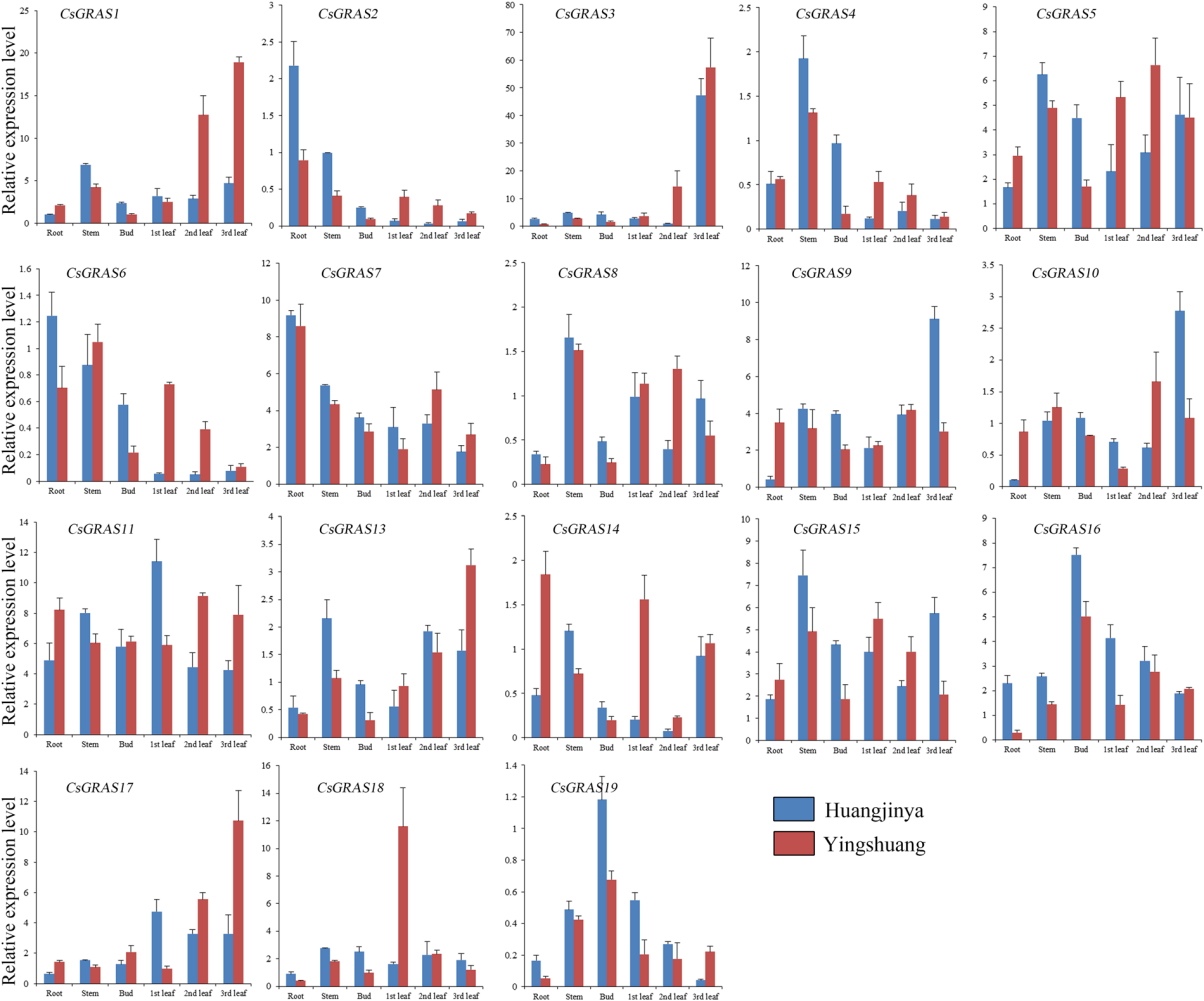
Tissue-specific expression profiles of *CsGRAS* genes in tea plant cultivars ‘Huangjinya’ and ‘Yingshuang’.

Results indicated that these *CsGRAS* genes were detected in all of the tissues of the two tea plant cultivars and exhibited variable transcript levels (Fig. 7). *CsGRAS1*, *3*,* 5*, *7*, *9*, *11*, *15*, *16*, *17*, and *18* were expressed at relatively high levels, whereas the other genes were expressed at low levels. Most of the *CsGRAS* genes presented similar tissue-specific expression patterns between two tea plant cultivars. For example, in two tea plant cultivars, *CsGRAS2* and *CsGRAS7* showed relatively high expression levels in the roots; *CsGRAS16* and *CsGRAS19* were abundantly expressed in the buds; *CsGRAS*3 was highly expressed in the third leaf; *CsGRAS8* was highly expressed in the stems and leaves and weakly expressed in the roots and buds. The transcript levels of *CsGRAS1* and *CsGRAS13* gradually increased in the first, second, and third leaves. Some differences also existed. For example, CsGRAS*10*showed the highest expression in the second leaf in ‘Yingshuang’ but was highly abundant in the third leaf in ‘Huangjinya’.

### Expression analysis of *CsGRAS* genes under GA treatment in two tea plant cultivars

**Figure 8:**
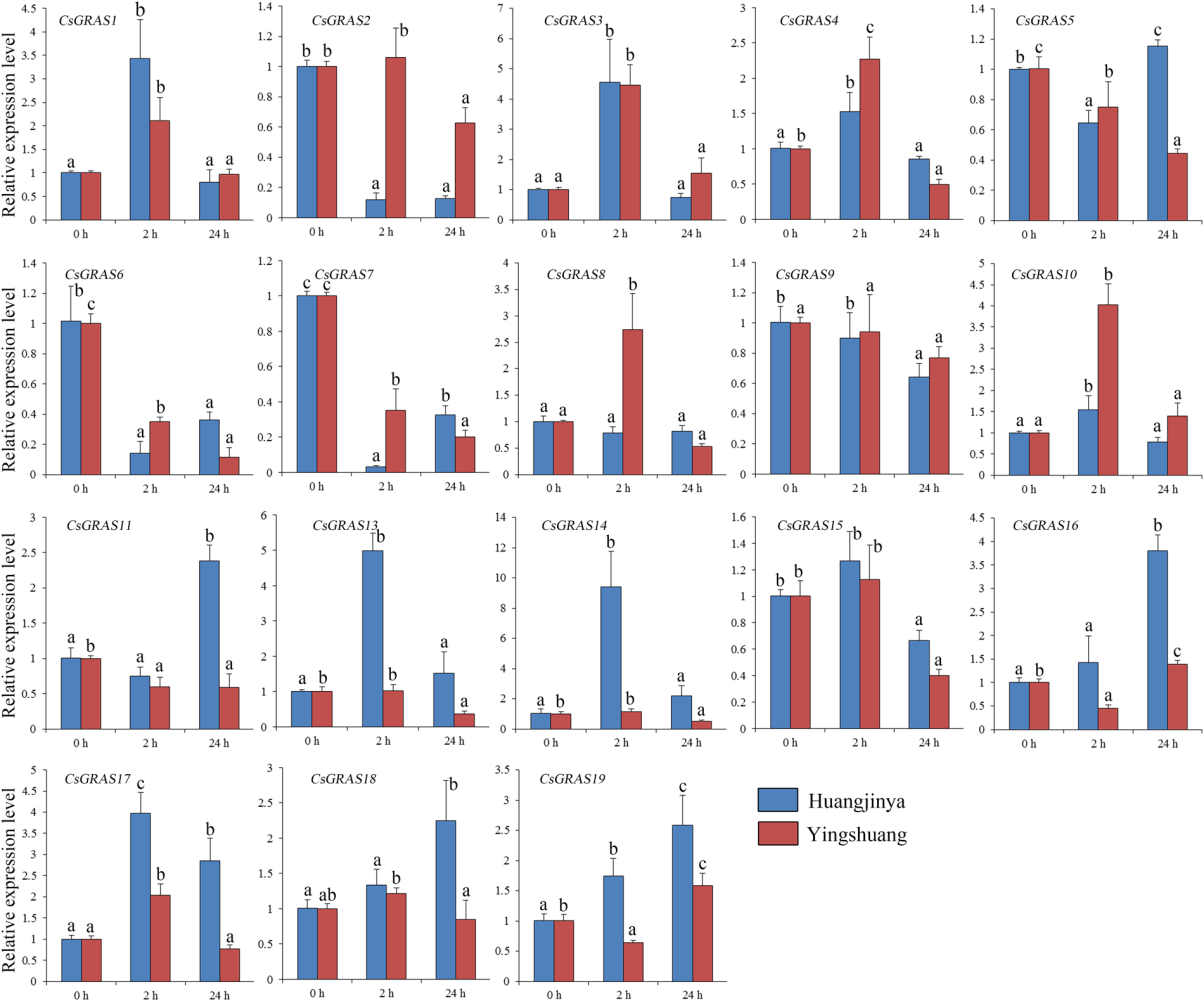
Expression patterns of *CsGRAS* genes in response to GA treatment in tea plant cultivars ‘Huangjinya’ and ‘Yingshuang’.

Considering that many GRAS proteins are involved in regulating GA response, we examined the response of* CsGRAS* genes to GA treatment in ‘Huangjinya’ and ‘Yingshuang’ through qRT-PCR. Under GA treatment (Fig. 8), three *CsGRAS* genes (*CsGRAS1*, *3*, and *17*) were obviously upregulated in both tea plant cultivars, whereas *CsGRAS2*,* 6*,* 7*, and *15*were obviously downregulated. Three *CsGRAS* genes (*CsGRAS11*,* 13*, and *14*) were mainly upregulated in ‘Huangjinya’ but downregulated in ‘Yingshuang’. *CsGRAS8* and *CsGRAS10* were mainly upregulated in ‘Yingshuang’ but showed a relatively stable expression level in ‘Huangjinya’.

### Expression analysis of *CsGRAS* genes under different abiotic treatments in two tea plant cultivars

The responses of *CsGRAS* genes to salt (200 mM NaCl), drought (20% PEG 6000), cold (4 °C), and heat (38 °C) treatments in ‘Huangjinya’ and ‘Yingshuang’ were examined through qRT-PCR to elucidate further the role of GRAS genes in tea plant during diverse environmental stresses. Except for *CsGRAS3*, all of the analyzed genes responded to at least one abiotic stress treatment. Although most *CsGRAS* genes exhibited a similar tendency in the two tea plant cultivars, some notable exceptions were also observed.

#### Salt treatment

**Figure 9:**
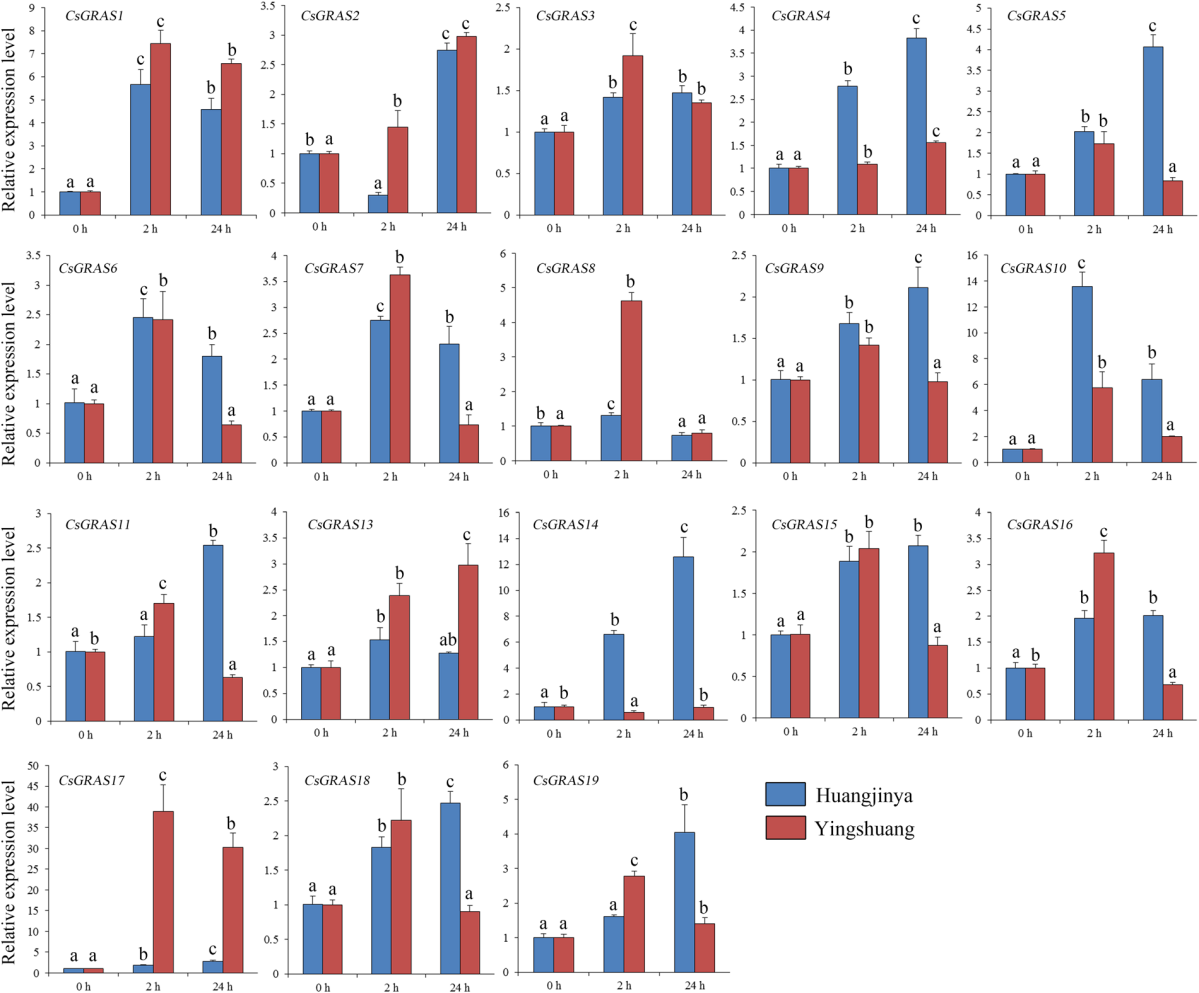
Expression patterns of *CsGRAS* genes in response to salt treatment in tea plant cultivars ‘Huangjinya’ and ‘Yingshuang’.

Under salt treatment (Fig. 9), 10 *CsGRAS* genes (*CsGRAS1*, *2*,* 6*, *7*, *10*, *15*, *16*, *17*, *18*, and *19*) showed obvious increases in both tea plant cultivars. Some *CsGRAS* genes (*CsGRAS4*, *5*, *8*, *9*, *11*, *13*, and *14*) were only increased in one tea plant cultivar. *CsGRAS1*, *10*,* 14*, and *17* were the most highly induced genes. *CsGRAS10* rapidly reached the peak at 2 h and then decreased at 24 h in both tea plant cultivars. *CsGRAS14* was highly induced in ‘Huangjinya’, whereas *CsGRAS17* was highly induced (approximately fortyfold) in ‘Yingshuang’.

**Figure 10:**
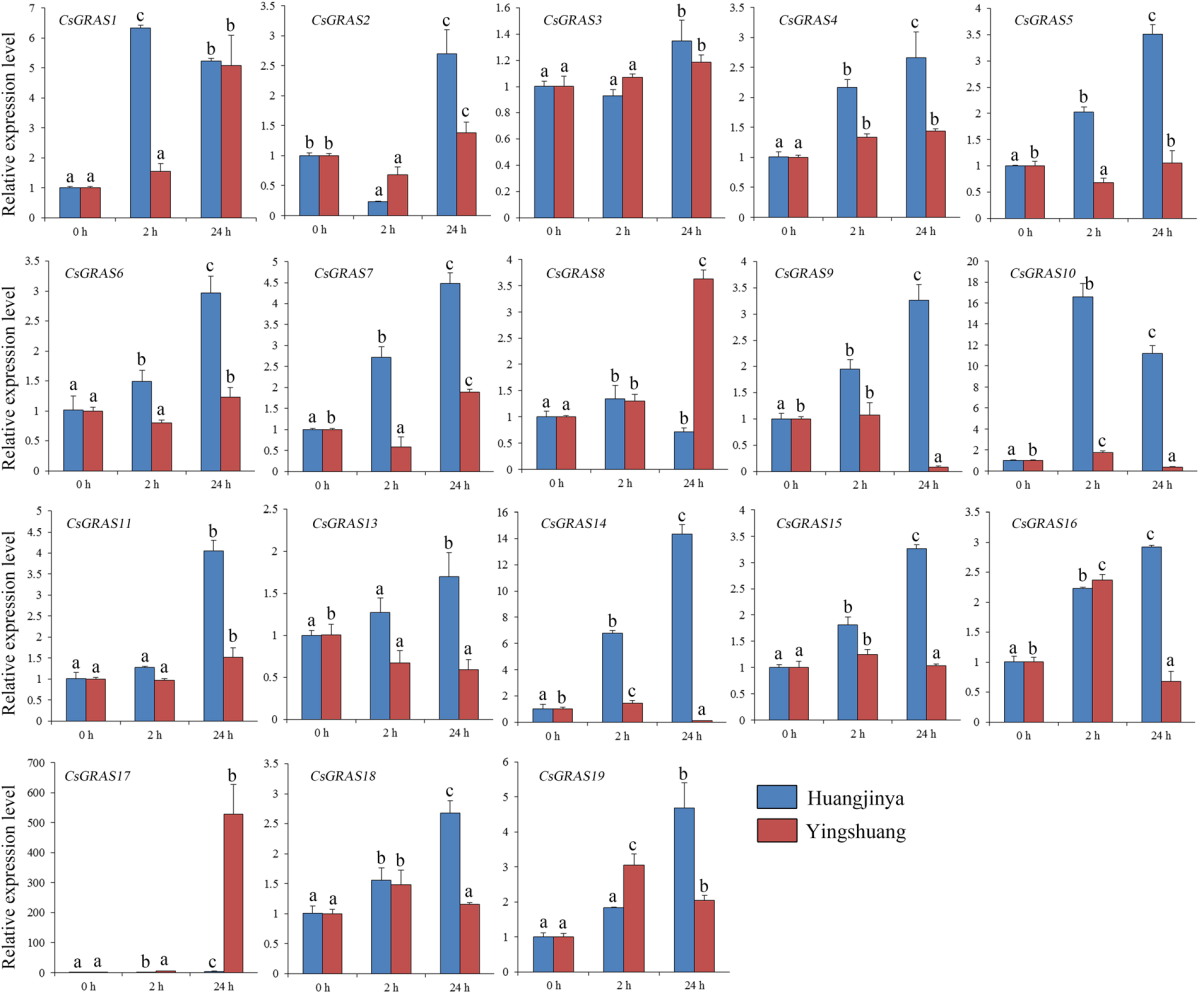
Expression patterns of *CsGRAS* genes in response to drought treatment in tea plant cultivars ‘Huangjinya’ and ‘Yingshuang’.

#### Drought treatment

Under drought treatment (Fig. 10), 10 *CsGRAS* genes (*CsGRAS1*, *16*, *17*, and *19*) were induced in both tea plant cultivars. Three *CsGRAS* genes (CsGRAS*9*, *10*, and *14*) were obviously upregulated in ‘Huangjinya’ but downregulated in ‘Yingshuang’. *CsGRAS4*, *5*, *6*, *7*, *11*, *15*, and *18* were increased in ‘Huangjinya’ but showed relatively consistent expression profiles in ‘Yingshuang’. *CsGRAS1*, *10*, *14*, and *17* were the most highly induced genes. *CsGRAS11* and *CsGRAS14* were highly induced in ‘Huangjinya’, whereas *CsGRAS17* was highly induced (over 300-fold at 24 h) in ‘Yingshuang’.

#### Low-temperature treatment

**Figure 11:**
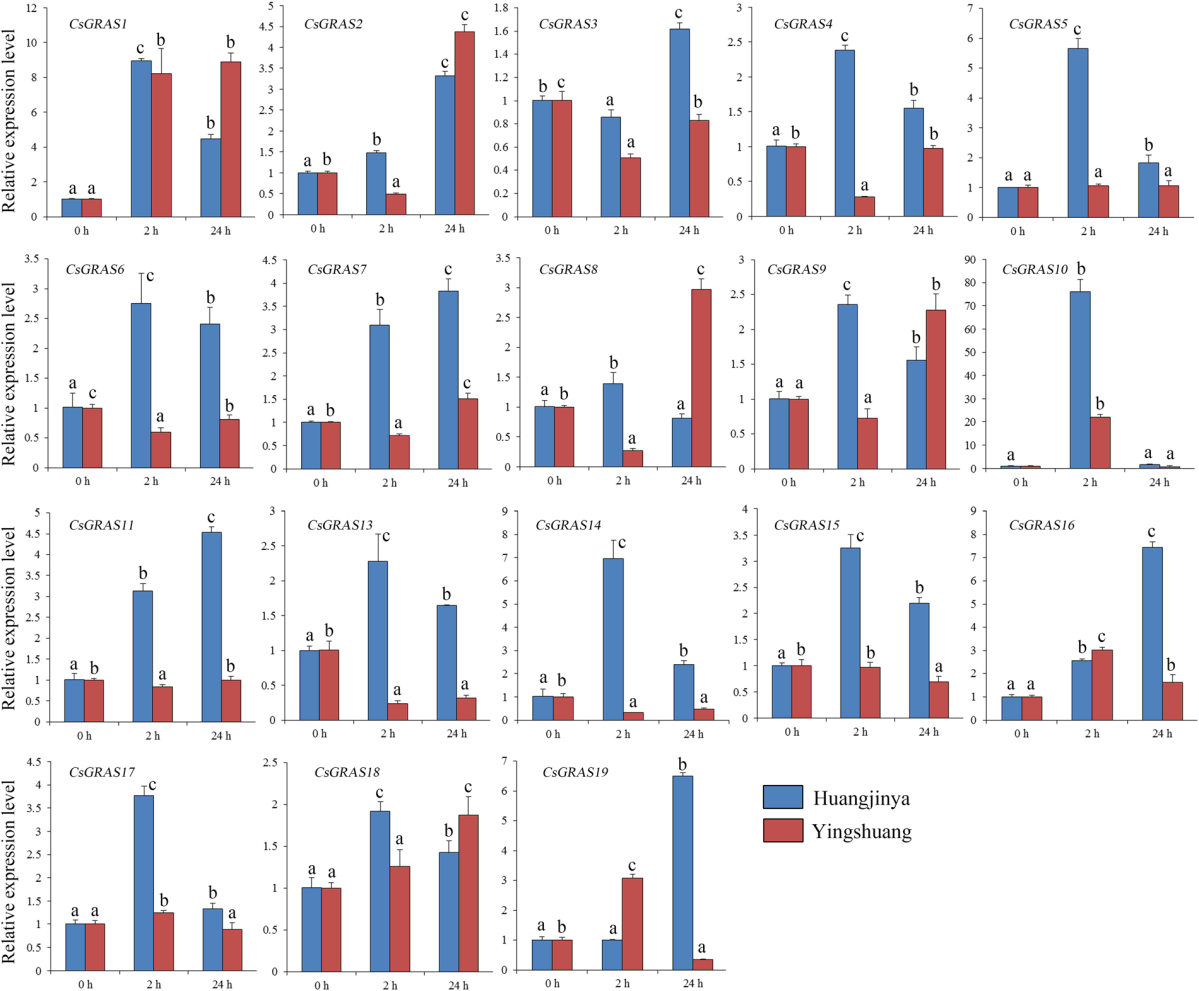
Expression patterns of *CsGRAS* genes in response to cold treatment in tea plant cultivars ‘Huangjinya’ and ‘Yingshuang’.

Under low-temperature treatment (Fig. 11), six *CsGRAS* genes (*CsGRAS1*, *2*, *9*, *10*,* 16*, and *19*) showed obvious increases in both tea plant cultivars. Six *CsGRAS* genes (*CsGRAS5*, *6*, *7*,* 11*, *15*, and *17*) were mainly upregulated in ‘Huangjinya’ but showed a relatively stable expression level in ‘Yingshuang’. *CsGRAS4*, *13*, and *14* were obviously increased in ‘Huangjinya’ but decreased in ‘Yingshuang’. *CsGRAS1* and *CsGRAS10* were the most highly induced genes. *CsGRAS1* and *CsGRAS10* were upregulated in both tea plant cultivars, of which *CsGRAS10* rapidly reached the peak at 2 h and then decreased at 24 h in both cultivars (over 75- and 20-fold, respectively).

#### High-temperature treatment

**Figure 12:**
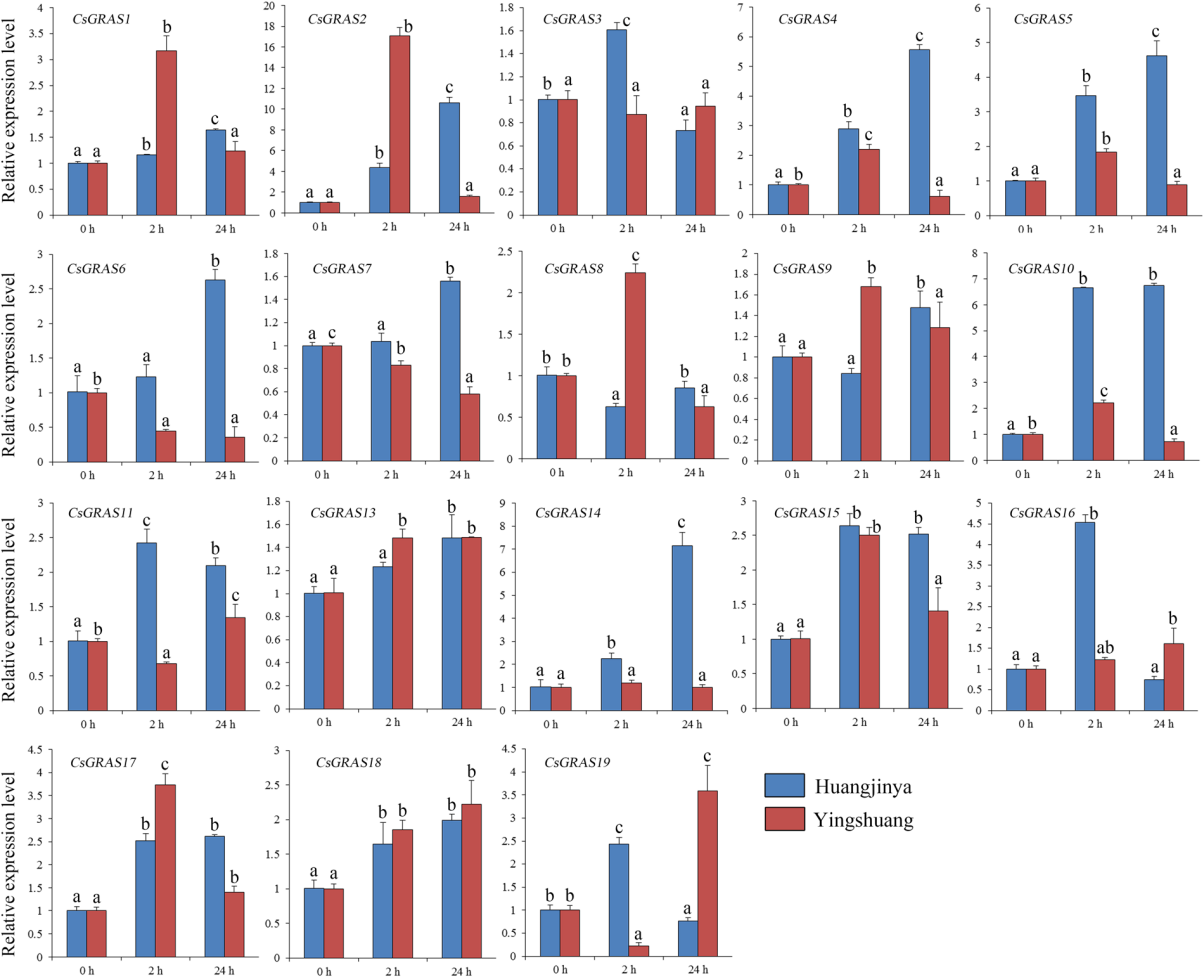
Expression patterns of *CsGRAS* genes in response to heat treatment in tea plant cultivars ‘Huangjinya’ and ‘Yingshuang’.

Under high-temperature treatment (Fig. 12), six *CsGRAS* genes (*CsGRAS* *2*, *4*, *10*, *15*, *17*, and *19*) were evidently upregulated in both tea plant cultivars. *CsGRAS1*, *5*, *8*, *11*, *14*, *16*, and *18* were increased in one tea plant cultivar. *CsGRAS2*, *10*, and *14*were the most highly induced genes. *CsGRAS2* was rapidly increased at 2 h and then decreased at 24 h in ‘Yingshuang’, whereas it reached the peak at 24 h in ‘Huangjinya’. *CsGRAS10* and *CsGRAS14* were mainly upregulated in ‘Huangjinya’ but showed a relatively stable expression level in ‘Yingshuang’.

In general, most *CsGRAS* genes were affected by drought, salt, and high and cold stresses. *CsGRAS1*, *10*, and *17* from subfamily AtPAT1 and *CsGRAS14* from subfamily LISCL were identified as highly induced genes.

## Discussion

In recent years, a large number of TFs have been identified and analyzed in various species through bioinformatics technology. Compared with that of other TFs families, the research progress of the GRAS family is relatively slower. To date, insufficient information is available about GRAS TFs in tea plant. In the present study, 52 *CsGRAS* genes were identified from tea plant genome database.

Gene loss and duplication events are the dominant driving forces in species evolution, supplying the raw materials for the generation of novel gene functions and promoting the formation of gene families. No GRAS family members were found in lower plants. However, a recent study has identified the GRAS gene family in bacteria and classified it under the Rossmann fold methyltransferase superfamily^26^. Although methyltransferase activity is possibly lacking in most GRAS proteins in plants, the GRAS proteins in plants still bind a similar substrate to those in bacteria^32^. Exon–intron organization analysis on several species, such as *Prunus mume*, Arabidopsis, rice, populous, and grapevine, reported that most GRAS genes either lack introns or possess only a single intron^27,30,32,35^. Thus, horizontal gene transfer from ancient prokaryote genomes of bacteria may explain the origin of plant GRAS genes.

Gene duplication and differentiation are the major pathways of origin for new genes and for the differentiation of gene function^52^. Members clustered in similar subgroups commonly exhibit functional similarities. Therefore, phylogenetic analysis could facilitate functional genomics. In the present study, we identified 135 GRAS proteins with full-length domain sequences from tea plant, Arabidopsis, and rice, and classified them into 13 subfamilies on the basis of sequence structures and phylogenetic relationships. All of the five predicted tea plant DELLA (CsGRAS11, -12, -16, -24, and 48) proteins together with other DELLA proteins (AtGAI, AtRGA, AtRGL1, AtRGL2, AtRGL3, and OsSLR1) were grouped into the DELLA subfamily. Some rice GRAS members (OsGRAS1 and OsGRAS27) that were similar to the other DELLA proteins but lacked the DELLA domain were also grouped into the DELLA subfamily. Previously, Liu *et al*. grouped OsGRAS1 and OsGRAS27 together with the other DELLA proteins into the DELLA subfamily^32^. The results are largely consistent with previous reports. Considerable bootstrapping value supports existed for many of the defined subfamilies in the unrooted tree, although some poor supporting values remained for several clusters. This result was expected because the GRAS protein sequences used in this study had an average of approximately 600 amino acid-lengths, a constraint imposed by a large number of substitutable residues.

Phylogenetic analysis revealed the following several pairs of closely related orthologous GRAS proteins between tea plant and Arabidopsis/rice: CsGRAS8 and AtSCL15, CsGRAS11 and AtRGA and AtGAI, CsGRAS2 and AtSCL3, CsGRAS19 and AtSCL28, CsGRAS20 and AtSCR, CsGRAS3 and AtSHR, CsGRAS1 and OsCIGR1, CsGRAS5 and AtSCL1, CsGRAS4 and AtSCL9, and CsGRAS10 and CsGRAS17 with AtSCL13. Orthologs generally retained similar functions. AtSCL3, which shows a high similarity to CsGRAS2, functions as a repressor of DELLA and controls root development through the GA pathway^53,54^. CsGRAS19, which shares a high similarity to OsGRAS29 (also known as DLT), is involved in brassinosteroid signaling^55^. CsGRAS3 and CsGRAS20 show high degrees of similarity to AtSHR and AtSCR, respectively, which are associated with root and shoot radial pattern formation^41–43^. CsGRAS10 and CsGRAS17 show high degrees of similarity to AtSCL13, which is a positive regulator of phytochrome B signaling^56^. The results suggest the possible functions of GRAS genes in tea plant.

The structural divergence of gene sequences has played vital roles in the evolution of gene families, which is an adaptive process for speciation and leads to the efficient use of natural resources or adaptation to unfavorable conditions. Comparisons of GRAS proteins between tea plant and Arabidopsis were conducted to characterize the GRAS sequences. Most GRAS proteins clustered into similar subgroups sharing similar motifs, suggesting their group-specific functions. Significant differences were observed among the different groups. For example, six motifs (motifs 17, 20, 21, 23, 25, and 27) were only present in subgroup LISCL; two motifs (motifs 24, and 28) were only found in subgroup AtPAT1, indicating their specific functions to other subgroup members. Among them, the DELLA (motif 15) and TVHVNP (motif 18) domains function in GA perception^29,51,57^. The distributions of conserved motifs also reflect the relations of different subgroups. For example, motif 6 was found and 11 was completely lost in subgroups AtPAT1 and LISCL. This result indicates the close evolutionary relationships between subgroups AtPAT1 and LISCL. Therefore, structural analysis also provides a clue to locate which subgroup of GRAS genes is the ancient origin^58^.

Type I and type II functional divergence between subfamilies of GRAS genes were estimated. Significant type I functional divergence was detected between any two subfamilies, suggesting that site-specific rate shift after gene duplication is the main force driving the functional divergence in the evolution of the GRAS family. Type II functional divergence between most subfamily pairs was smaller than 0, whereas the standard errors were relatively high, suggesting that the GRAS gene family may adopt type II in different degrees. In the present study, the functional divergence of GRAS genes was not closely correlated with expression profiling in different organs and samples under different abiotic stresses. It may contribute potentially to gene expression profiling accumulation.

The functional annotations of CsGRAS proteins were obtained from the well-characterized biological function of similar genes. It provided a functional context for the *CsGRAS* genes in tea plant with modules. *CsGRAS* genes were predicted to have diverse functions and involved in various physiological processes, such as responses to abiotic and biotic stresses, hormones, tissue development, and secondary metabolites.

The gene expression levels could provide critical clues to assess their possible functions. On the basis of previous transcriptome sequencing data, the transcript levels of the *CsGRAS* genes among different tea plant cultivars under normal conditions were investigated. In consideration these cultivars were obtained from different regions of China^7^, the transcription levels were varied among the four tea plant cultivars. *CsGRAS7* and two DELLA genes (*CsGRAS11* and *CsGRAS12*) were stable and highly expressed in the four cultivars, indicating their critical roles in tea leaf development and metabolite biosynthesis.

Tissue-specific genes promote the development of particular organs or tissues, and tissue-specific expressions are helpful in defining the precise nature and function of genes. Eighteen tested *CsGRAS* genes widely exist in the tissues (the root, stem, bud, and 1st, 2nd and 3rd leaf) between the two tea plant cultivars (‘Huangjinya’ and ‘Yingshuang’). Some results showed great error bars in expression levels, which may mainly because the differences among biological individuals. The expression patterns of different genes varied greatly; some genes showed similar tissue-specific expression patterns between two tea plant cultivars. The transcript levels of *CsGRAS1* and *CsGRAS13* gradually increased in the 1st, 2nd and 3rd leaf, indicating those two genes may function in the later development of different tissues of tea plant. It is observed that the expression levels of most of the *CsGRAS* genes in 1st, 2nd and 3rd leaf were higher for ‘Yingshuang’ compared to ‘Huangjinya’. That may mainly because *CsGRAS* genes play greater roles in ‘Yingshuang’ compared to ‘Huangjinya’ during leaf development. The DELLA gene *CsGRAS11* showed high expression levels in all tested tissues, which is consistent with DELLA genes that serve as the main signaling hub in the regulation of multifarious growth and developmental processes^59,60^. The DELLA gene *CsGRAS16* and DLT gene *CsGRAS19* showed similar expression patterns and relatively higher expression levels in the buds than in the other tissues, indicating that these genes play a role in bud development. *CsGRAS2* and *CsGRAS7* showed high transcript levels in the roots. Previous studies reported that the *CsGRAS2* homolog *AtSCL3* regulates root development through the GA pathway^53,54^.

GA is involved in multiple aspects of plant growth and development, such as seed development and germination, stem elongation, and flower development. The prediction of the functional interaction network indicated that the GRAS proteins act as important components of the GA signaling pathway. Expression analysis indicated that most GRAS genes were induced or inhibited in at least one tea plant cultivar. In Arabidopsis, DELLA proteins act as repressors of GA-responsive plant growth. Two DELLA genes (*CsGRAS11* and *CsGRAS16*) were affected by GA treatment in tea plant. AtSCL3 represses DELLA and controls root development through the GA pathway. *CsGRAS2* is an orthologous gene in tea plant, and it is evidently downregulated under GA treatment.

**Figure 13:**
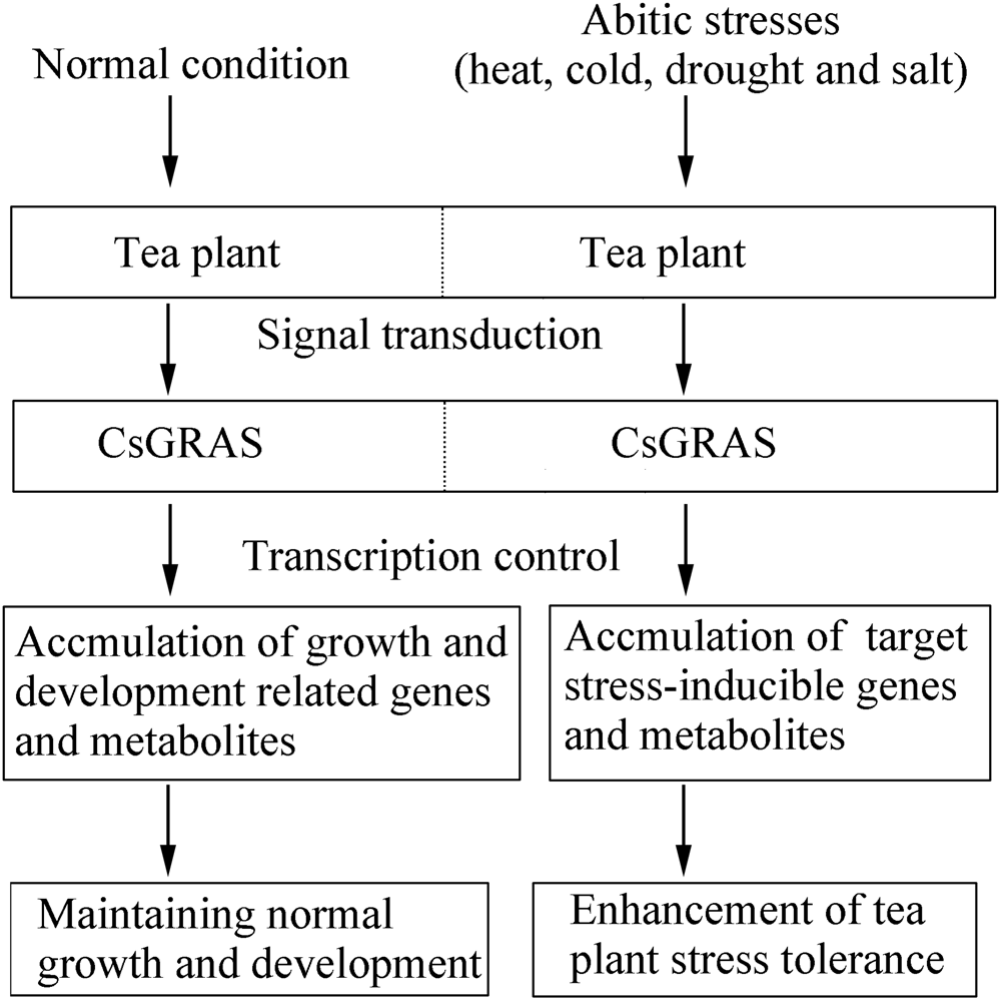
A potential model of transcriptional regulation of GRAS TFs in tea plant.

Harmful environmental conditions, such as salt, drought, cold, and heat, result in irreversible damages to the growth and development of tea plant. GRAS genes reportedly play potential regulatory roles in plant response to abiotic stresses (Fig. 13)^33,61–64^. However, information about this family in tea plant is currently lacking. In the present study, expression analysis indicated that most GRAS genes in tea plant could be affected by one or more various stress treatments, suggesting that *CsGRAS* family genes play important roles in the response to adversity stresses. Some expression trend differences exist between the cultivars ‘Huangjinya’ and ‘Yingshuang’. Most of *CsGRAS* genes studied have shown higher levels of expression in ‘Huangjinya’ compared to ‘Yingshuang’ under salt treatment, indicating *CsGRAS* genes may have more important roles in ‘Huangjinya’ than that in ‘Yingshuang’ under salt stress. The expression pattern of *CsGRAS6* under heat treatment has shown exactly opposite pattern in the two tea plant cultivars. Those different trends of expression of *CsGRAS* genes maybe involved in the response to abiotic stress in the two tea plant cultivars. Several highly induced genes were identified in subfamilies AtPAT1 (*CsGRAS1, 10*, and *17*) and LISCL (*CsGRAS14*). Two members of the AtPAT1 subfamily genes from rice, *CIGR1* and *CIGR2*, are involved in GA and stress response^65^. *AtSCL13*, an AtPAT1 subfamily gene from Arabidopsis, responds to various abiotic stresses and phytohormones^56^. The rice gene *CIGR1* is orthologous to *CsGRAS1*, and the Arabidopsis gene *AtSCL13* is orthologous to *CsGRAS10* and *CsGRAS17*. Likewise, *CsGRAS14* shares a strong sequence similarity to *AtSCL14*, which plays vital roles in the activation of stress-inducible promoters^66^. A previous expression analysis on *Castor Beans* indicated that LISCL and AtPAT1 subfamily genes respond to all of the four abiotic stresses (drought, salt, cold, and heat)^34^. These genes could be preferentially utilized for further plant functional studies. Several other genes respond to abiotic stresses. *OsGRAS23*, an AtSCL3 subfamily gene from rice, is involved in drought stress response through regulating the expression of stress responsive genes^67^. *CsGRAS2*, also an AtSCL3 subfamily gene in tea plant, is evidently upregulated under drought, salt, heat, and cold treatments, especially under heat stress. DELLA proteins participate in multiple abiotic stresses, such as low temperature and phosphate starvation^68,69^. The DELLA member *CsGRAS16* is upregulated under all of the four abiotic treatments in both tea plant cultivars, whereas the expression of the *CsGRAS11* genes dramatically changes under the four abiotic stress treatments in ‘Huangjinya’ but is relatively stable in ‘Yingshuang’.

Till now, tea plant transgenic system was not efficient. It was difficult to obtained transgenic tea plants. For further verify the function of those *CsGRAS* genes of tea plant, we shall carry out the *CsGRAS* genes overexpression and gene knockout using tea plant system, Arabidopsis or other model plants by transgenic method. In the future, using new and modified Sanger and next-generation sequencing techniques, more quality tea plant genome data shall be performed. We could integrate various genomics, transcriptomics, proteomics, and metabolomics to understand the *CsGRAS* genes function of tea plant.

## Conclusion

The GRAS TF family has been characterized in several plant species and associated with various critical development and physiological processes. However, information about this gene family in tea plant is lacking. In this work, 52 genes encoding CsGRAS family TFs were identified from tea plant. The phylogenetic relationship, conserved motif composition, and functional divergence of these *CsGRAS* genes were systematically analyzed and compared, which provided insights into the functional diversity of the GRAS gene family. The expression analyses of *CsGRAS* genes among different tissues (root, stem, bud, first leaf, second leaf, and third leaf), GA treatment, and abiotic stresses (salt, drought, heat, and cold) provide valuable information about the gene functions of this TF family in tea plant. This study could serve as a reference for future function investigations and molecular breeding of tea plant.

## Materials and Methods

### Retrieval of putative GRAS genes of tea plant

All candidate *CsGRAS* genes were derived from the tea plant genome database^18^. The acquired amino acid sequences were analyzed using the BLASTP search at NCBI (http://blast.ncbi.nlm.nih.gov/Blast.cgi). Only the sequences with full-length GRAS domain were considered as CsGRAS proteins and used for further analyses. HMMs of the selected CsGRASs were analyzed using the Pfam database (http://pfam.sanger.ac.uk)^70^. The chemical and physical characteristics of the CsGRAS proteins were predicted by ProtParam (http://web.expasy.org/protparam/). To confirm the subcellular localization of the identified CsGRAS proteins, protein sequences were predicted using WoLF PSORT (http://wolfpsort.org). TMHHM server v2.0 (http://www.cbs.dtu.dk/services/TMHMM/) was used to predict the membrane-bound CsGRAS members. The GRAS family TF databases of other species were downloaded from the plant TFDB database (http://planttfdb.cbi.edu.cn/)^71^.

### Phylogenetic and evolutionary analyses of the GRAS TFs

Multiple sequence alignment of the identified GRAS protein sequences was performed using the ClustalX1.83 program with default parameters and adjusted manually^72^. The sequence alignments were also used for the subsequent phylogenetic analysis. Phylogenetic trees were generated with MEGA5.02 software by using the NJ, ME, and ML algorithms, and the reliability of the obtained trees was assessed with a bootstrap value of 1000^73,74^. Functional divergence analysis was performed using the DIVERGE (v2.0) software with the algorithm based on ML procedures^47,50^. MEME v4.11.1 (http://meme-suite.org/tools/meme) was employed to identify the conserved motifs with default parameters, except for the maximum number of motifs (30). The functional interacting networks of proteins were constructed using STRING software with the confidence parameter set at 0.15 threshold^75^.

### RNA-seq data analysis

The expression profiles of *CsGRAS* genes in the four tea plant cultivars were assessed using transcriptome sequencing data, which was previously generated and analyzed by Wu *et al*.^7^. The tea plant samples used included mid-leaf ‘Yunnanshilixiang’ (Tea_T1) from Yunnan Province, small-leaf ‘Chawansanhao’ (Tea_T2) from Jiangsu Province, large-leaf ‘Ruchengmaoyecha’ (Tea_T3) from Hunan Province, and small-leaf ‘Anjibaicha’ (Tea_T4) from Zhejiang Province. These four tea plant samples exhibited different environmental adaptation modes and leaf sizes^7^. RPKM values were retrieved and normalized to estimate gene expression values. Heat maps were generated, and hierarchical clustering was conducted using HemI1.0 software (http://hemi.biocuckoo.org/faq.php).

### Tea plant materials and stress treatments

In China, ‘Huangjinya’ and ‘Yingshuang’ are important tea plant cultivars that share several identical traits, such as high yield and good quality. ‘Huangjinya’ is a light-sensitive albino cultivar that shows potential for processing high-quality green tea even in summer but exhibits weak stress resistance^76,77^. ‘Yingshuang’ possesses appropriate phenol ammonia content and good resistance to abiotic stresses, especially cold stress. Two-year-old vegetative propagated cuttings of tea plants, such as ‘Huangjinya’ and ‘Yingshuang’, were grown in a chamber at the Tea Science Research Institute of Nanjing Agricultural University (Nanjing, China). The seedlings were grown in pots containing a mixture of perlite, vermiculite, and sphagnum (ratio, 1:2:3). The chamber conditions were 23 °C temperature, 14/10 h day/night period, and 70% relative humidity for subsequent stress treatments. To determine the tissue-specific expression patterns of *CsGRAS* genes, the roots, stems, buds, and first, second, and third leaves were collected from ‘Huangjinya’ and ‘Yingshuang’ under normal conditions, rapidly frozen in liquid nitrogen, and then stored at −80 °C for RNA extraction. Those two tea plant cultivars were subject to different treatments, such as cold, heat, high salinity, drought, and GA treatments. The tea plants were treated according to the procedure basis on the previous reports with a slight modification^77,78^. In brief, for extreme temperature treatments, the seedlings were transferred to another chamber and maintained at 4 °C and 38 °C. For drought and salt treatments, the seedlings were irrigated with PEG6000 (20%) and NaCl (200 mM) solutions, respectively. For GA treatment, the seedlings were sprayed with GA3 (1 mM). After 0, 2 (short time), and 24 h (long time) treatments, the young leaves of tea plant were collected, rapidly frozen in liquid nitrogen, and then stored at −80 °C.

### RNA isolation and qRT-PCR analysis

The total RNA was isolated from plant samples using the Quick RNA Isolation Kit (Huayueyang, Beijing, China) in accordance with the manufacturer’s protocol. The purified RNA (1.0 µg) was reverse-transcribed into cDNA in 20 µL of reaction volume using a PrimeScript RT reagent kit (TaKaRa, Dalian, China). The cDNA reaction mixture was diluted 20-fold using deionized water for fluorescence detection.

The specific primers for tea plant *CsGRAS* genes were designed from the non-conserved N-terminal region using Primer Premier 5.0 software. Primer specificity was confirmed by the melting curve with a single sharp peak and the electrophoresis pattern with a single amplicon and the correct predicted length. The accuracy of PCR amplified bands were validated by PCR paired-end sequencing, and completed by the GenScript Corporation (Nanjing, China) (Figs S5 and S6).The *Csactin* gene served as an internal control to normalize the expression levels^78,79^. All primer sequences are presented in Table 3. qRT-PCR assays were completed on the Bio-Rad CFX96 fluorescence quantitative PCR platform with the following program: 95 °C for 3 min; 40 cycles of 95 °C for 5 s for denaturation and 58 °C for 30 s for annealing and extension; and 61 cycles 65 °C for 10 s for melting curve analysis. Each reaction was conducted in a 20 µL reaction mixture containing 2 µL of diluted template cDNA, 0.4 µL of each specific primer, 10 µL of SYBR Premix *Ex-Taq* (TaKaRa, Dalian, China), and 7.2 µL of ddH_2_O. The experiments were independently repeated thrice, and relative expression levels were measured using the 2^−ΔΔCt^ method^80^. Significant differences were detected using Duncan’s multiple-range test at the 0.05 level with SPSS 17.0 software.

**Table 3:** Sequences of primers used in qRT-PCR.

Target Gene	Forward primer sequence (5′ → 3′)	Reverse primer sequence (5′ → 3′)
*CsGRAS1*	GCCAGATTTTTGGATACGG	ACTCAATGTGGTAAGGGTCTCA
*CsGRAS2*	AGAGATCATCATCAGTGAGTTCG	CCACGGAAATGGTGAACC
*CsGRAS3*	ATCAGTCCTTCAACTCCAGC	GGTGGTAAGGGTAGTAGTGCT
*CsGRAS4*	CAGATTAGAAGTCCTCCAGTGTA	TTCCTGAGGTGAAAGTCCAT
*CsGRAS5*	AGCGAGAGTTATGACCCGA	GATACCCATCCTGATGCCTTA
*CsGRAS6*	CCTCAACCCCAATCCCA	CAACCCTGACGACTCTCCTT
*CsGRAS7*	CAGAACAGAACCAGGGGGA	CAAATGAGGATTCAAGCGAG
*CsGRAS8*	GAAAGTTCCGTTTATCTCAGCC	CGACTGTAACAAAGCATCCGA
*CsGRAS9*	GCCCTACAAGAAACTGAGAAA	AGTCAAATGACAACTGCGAA
*CsGRAS10*	ACATCGCAGGAACATCAAAG	TCCGACAGGTATGACTGAGAAC
*CsGRAS11*	AGCATCACCAGCACTACCC	TGTTCCAGCTTCTGAGCAAC
*CsGRAS13*	GTGGACGGTGGCTCTAATG	AAGATGGTGTTGATAGGGTTGT
*CsGRAS14*	AGCAGTCAGCAGTTTTTATGGA	ACTTCCGTTTTGTTGCCTTC
*CsGRAS15*	GCCAGAGGTTGAGTCCTATCC	GTCCGTCAAGCAAATCCC
*CsGRAS16*	GTGGGACCCGCATTAGAT	ACCGATAACTGCCGAGGA
*CsGRAS17*	ATGGGAGCCAAGCAACC	GACTGAGAACCTTGTGGTGAAA
*CsGRAS18*	GGACTCATTTCCAGCAACG	AAGTTCACGCAGCTTGTGTC
*CsGRAS19*	CCTGTATGAAGAGGGCTAAGAG	CTCAAAACTTGGCTGAAACC
*CsActin*	GATTCCGTTGCCCTGAAGTCCT	CCTTGCTCATACGGTCTGCGATA

## Electronic supplementary material

Supplementary Figures

Supplementary Tables

## References

[1] Mondal TK, Bhattacharya A, Laxmikumaran M, Ahuja PS (2004). Recent Advances of Tea (*Camellia Sinensis) Biotechnology*. Plant Cell Tissue & Organ Culture.

[2] Weidong Wang, H. X. *et al.* Transcriptomic Analysis Reveals the Molecular Mechanisms of Drought-Stress-Induced Decreases in *Camellia sinensis* Leaf Quality. *Front. Plant Sci*., 10.3389/fpls.2016.00385 (2016). 10.3389/fpls.2016.00385 PMC4811933 27066035

[3] Mukhopadhyay M, Mondal TK, Chand PK (2016). Biotechnological advances in tea (*Camellia sinensis* [L.] O. Kuntze): a review. Plant Cell Reports.

[4] Weng H (2017). Tea Consumption and Risk of Bladder Cancer: A Dose-Response Meta-Analysis. Frontiers in Physiology.

[5] Fang WP (2014). Varietal identification of tea (*Camellia sinensis*) using nanofluidic array of single nucleotide polymorphism (SNP) markers. *Horticulture*. Research.

[6] Chen Y, Yu M, Jie X, Chen X, Shi J (2009). Differentiation of eight tea (*Camellia sinensis) cultivars in China by elemental fingerprint of* their leaves. Journal of the Science of Food & Agriculture.

[7] Wu ZJ, Li XH, Liu ZW, Xu ZS, Zhuang J (2014). De novo assembly and transcriptome characterization: Novel insights into catechins biosynthesis in Camellia sinensis. BMC Plant Biology.

[8] Das, A., Das, S. & Mondal, T. K. Identification of Differentially Expressed Gene Profiles in Young Roots of Tea [*Camellia sinensis* (L.) O. Kuntze] Subjected to Drought Stress Using Suppression Subtractive Hybridization. *Plant Molecular Biology Reporter*, 1088–1101 (2012).

[9] Li XW (2010). A novel cold-regulated gene from Camellia sinensis, CsCOR1, enhances salt- and dehydration-tolerance in tobacco. Biochemical & Biophysical Research Communications.

[10] Vyas D, Kumar S (2005). Tea (Camellia sinensis (L.) O. Kuntze) clone with lower period of winter dormancy exhibits lesser cellular damage in response to low temperature. Plant Physiology & Biochemistry.

[11] Zhou Y (2017). Molecular cloning and characterization of galactinol synthases in Camellia sinensis with different responses to biotic and abiotic stressors. Journal of Agricultural & Food Chemistry.

[12] Zhang Y (2014). Identification and characterization of cold-responsive microRNAs in tea plant (*Camellia sinensis*) and their targets using high-throughput sequencing and degradome analysis. Bmc Plant Biology.

[13] Xin-Chao W (2013). Global transcriptome profiles of *Camellia sinensis* during cold acclimation. BMC Genomics.

[14] Li CF (2016). Biochemical and transcriptomic analyses reveal different metabolite biosynthesis profiles among three color and developmental stages in â ˜Anji Baichaâ™ (*Camellia sinensis*). Bmc Plant Biology.

[15] Li CF (2015). Global transcriptome and gene regulation network for secondary metabolite biosynthesis of tea plant (*Camellia sinensis*). Bmc Genomics.

[16] Zhang, Q., Liu, M. & Ruan, J. Integrated Transcriptome and Metabolic Analyses Reveals Novel Insights into Free Amino Acid Metabolism in Huangjinya Tea Cultivar. *Frontiers in Plant Science* **8** (2017). 10.3389/fpls.2017.00291 PMC5337497 28321230

[17] Ji HG (2017). Metabolic phenotyping of various tea (*Camellia sinensis* L.) cultivars and understanding of their intrinsic metabolism. Food Chemistry.

[18] Xia EH (2017). The Tea Tree Genome Provides Insights into Tea Flavor and Independent Evolution of CaffeineBiosynthesis. Molecular Plant.

[19] Yamaguchishinozaki K, Shinozaki K (2006). Transcriptional regulatory networks in cellular responses and tolerance to dehydration and cold stresses. Annual Review of Plant Biology.

[20] Zhuang J, Zhang J, Hou X-L, Wang F, Xiong A-S (2014). Transcriptomic, Proteomic, Metabolomic and Functional Genomic Approaches for the Study of Abiotic Stress in Vegetable Crops. Critical Reviews in Plant Sciences.

[21] Silva DCD (2016). Transcriptome analyses of the Dof-like gene family in grapevine reveal its involvement in berry, flower and seed development. Horticulture Research.

[22] Slobodan R, Diego RP, Ingo D, Bernd MR (2007). PlnTFDB: an integrative plant transcription factor database. BMC Bioinformatics.

[23] Wu ZJ (2015). Transcriptome-based discovery of AP2/ERF transcription factors related to temperature stress in tea plant (*Camellia sinensis)*. Functional & Integrative Genomics.

[24] Pysh, L. D., Wysockadiller, J. W., Camilleri, C., Bouchez, D. & Benfey, P. N. The GRAS gene family in Arabidopsis: sequence characterization and basic expression analysis of the\textit SCARECROW-LIKE genes. (1999). 10.1046/j.1365-313x.1999.00431.x 10341448

[25] Bolle C (2004). The role of GRAS proteins in plant signal transduction and development. Planta.

[26] Zhang, D., Iyer, L. M. & Aravind, L. *Bacterial GRAS domain proteins throw new light on gibberellic acid response mechanisms*. (Oxford University Press, 2012). 10.1093/bioinformatics/bts464 PMC3463117 22829623

[27] Tian C, Wan P, Sun S, Li J, Chen M (2004). Genome-Wide Analysis of the GRAS Gene Family in Rice and Arabidopsis. Plant Molecular Biology.

[28] Silverstone AL, Ciampaglio CN, Sun T (1998). The Arabidopsis RGA gene encodes a transcriptional regulator repressing the gibberellin signal transduction pathway. Plant Cell.

[29] Itoh H, Ueguchi-Tanaka M, Sato Y, Ashikari M, Matsuoka M (2002). The gibberellin signaling pathway is regulated by the appearance and disappearance of SLENDER RICE1 in nuclei. Plant Cell.

[30] Lu J, Wang T, Xu Z, Sun L, Zhang Q (2015). Genome-wide analysis of the GRAS gene family in *Prunus mume*. Molecular Genetics and Genomics.

[31] Song XM (2014). Genome-wide analysis of the GRAS gene family in Chinese cabbage (*Brassica rapassp*. pekinensis). Genomics.

[32] Liu X, Widmer A (2014). Genome-wide Comparative Analysis of the GRAS Gene Family in Populus, Arabidopsis and Rice. Plant Molecular Biology Reporter.

[33] Huang W, Xian Z, Xia K, Tang N, Li Z (2015). Genome-wide identification, phylogeny and expression analysis of GRAS gene family in tomato. BMC Plant Biology.

[34] Wei X (2016). Genome-Wide Identification, Evolutionary Analysis, and Stress Responses of the GRAS Gene Family in Castor Beans. International Journal of Molecular Sciences.

[35] Jérôme G, Patricia AR, Teixeira RT, Martinez-Zapater JM, Fortes AM (2016). Structural and Functional Analysis of the GRAS Gene Family in Grapevine Indicates a Role of GRAS Proteins in the Control of Development and Stress Responses. Frontiers in Plant Science.

[36] Peng J (1997). TheArabidopsis GAI gene defines a signaling pathway that negatively regulates gibberellin responses. Genes & Development.

[37] Wen CK, Chang C (2002). Arabidopsis RGL1 encodes a negative regulator of gibberellin responses. Plant Cell.

[38] Li X (2003). Control of tillering in rice. Nature.

[39] Schumacher K, Schmitt T, Rossberg M, Schmitz G, Theres K (1999). The Lateral suppressor (Ls) gene of tomato encodes a new member of the VHIID protein family. Proceedings of the National Academy of Sciences of the United States of America.

[40] Greb T (2003). Molecular analysis of the LATERAL SUPPRESSOR gene in Arabidopsis reveals a conserved control mechanism for axillary meristem formation. Genes & Development.

[41] Di LL (1996). The SCARECROW gene regulates an asymmetric cell division that is essential for generating the radial organization of the Arabidopsis root. Cell.

[42] Helariutta Y (2000). The SHORT-ROOT gene controls radial patterning of the Arabidopsis root through radial signaling. Cell.

[43] Sabatini S, Heidstra R, Wildwater M, Scheres B (2003). SCARECROW is involved in positioning the stem cell niche in the Arabidopsis root meristem. Genes & Development.

[44] Bolle C, Koncz C, Chua NH (2000). PAT1, a new member of the GRAS family, is involved in phytochrome A signal transduction. Genes & Development.

[45] Torres-Galea P, Hirtreiter B, Bolle C (2013). Two GRAS proteins, Scarecrow-LIKE21 and Phytochrome A Signal Transduction1, function cooperatively in phytochrome A signal transduction. Plant Physiology.

[46] Hirsch S (2009). GRAS proteins form a DNA binding complex to induce gene expression during nodulation signaling in Medicago truncatula. Plant Cell.

[47] Gu X (1999). Statistical methods for testing functional divergence after gene duplication. Molecular Biology & Evolution.

[48] Gu X (2001). Maximum-likelihood approach for gene family evolution under functional divergence. Molecular Biology & Evolution.

[49] Gu X, Velden KV (2002). DIVERGE: phylogeny-based analysis for functional–structural divergence of a protein family. Bioinformatics.

[50] Gu X (2006). A Simple Statistical Method for Estimating Type-II (Cluster-Specific) Functional Divergence of Protein Sequences. Molecular Biology & Evolution.

[51] Dill A, Sun T (2001). Synergistic derepression of gibberellin signaling by removing RGA and GAI function in *Arabidopsis thaliana*. Genetics.

[52] Song, A. *et al*. Transcriptome-Wide Identification and Expression Profiling of the DOF Transcription Factor Gene Family in *Chrysanthemum morifolium*. *Frontiers in Plant Science* **7** (2016). 10.3389/fpls.2016.00199 PMC4763086 26941763

[53] Heo JO, Estelle M (2011). Funneling of gibberellin signaling by the GRAS transcription regulator scarecrow-like 3 in the Arabidopsis root. Proceedings of the National Academy of Sciences.

[54] Zhang ZL (2011). Scarecrow-Like 3 promotes gibberellin signaling by antagonizing master growth repressor DELLA in Arabidopsis. Proceedings Of the National Academy Of Sciences Of the United States Of America.

[55] Tong H (2009). Dwarf and Low-Tillering, a new member of the GRAS family, plays positive roles in brassinosteroid signaling in rice. Plant Journal for Cell & Molecular Biology.

[56] Torresgalea P, Huang LF, Chua NH, Bolle C (2006). The GRAS protein SCL13 is a positive regulator of phytochrome-dependent red light signaling, but can also modulate phytochrome A responses. Molecular Genetics and Genomics.

[57] Fu X, Harberd NP (2002). Gibberellin-mediated proteasome-dependent degradation of the barley DELLA protein SLN1 repressor. Plant Cell.

[58] Li MY (2016). Genomic identification of WRKY transcription factors in carrot (*Daucus carota*) and analysis of evolution and homologous groups for plants. Scientific Reports.

[59] Bolle, C. *Functional Aspects of GRAS Family Proteins* (2016).

[60] Wild M (2012). The Arabidopsis DELLA RGA-LIKE3 is a direct target of MYC2 and modulates jasmonate signaling responses. Plant Cell.

[61] Yang M, Yang Q, Fu T, Zhou Y (2011). Overexpression of the *Brassica napus* BnLAS gene in Arabidopsis affects plant development and increases drought tolerance. Plant Cell Reports.

[62] Yuan Y (2016). Overexpression of VaPAT1, a GRAS transcription factor from Vitis amurensis, confers abiotic stress tolerance in Arabidopsis. Plant Cell Reports.

[63] Ma HS (2010). The salt- and drought-inducible poplar GRAS protein SCL7 confers salt and drought tolerance in *Arabidopsis thaliana*. Journal of Experimental Botany.

[64] Galovic V, Orlovic S, Fladung M (2015). Characterization of two poplar homologs of the GRAS/SCLgene, which encodes a transcription factor putatively associated with salt tolerance. Iforest.

[65] Day RB (2004). Two Rice GRAS Family Genes Responsive to N -Acetylchitooligosaccharide Elicitor are Induced by Phytoactive Gibberellins: Evidence for Cross-Talk Between Elicitor and Gibberellin Signaling in Rice Cells. Plant Molecular Biology.

[66] Fode B, Siemsen T, Thurow C, Weigel R, Gatz C (2008). The Arabidopsis GRAS protein SCL14 interacts with class II TGA transcription factors and is essential for the activation of stress-inducible promoters. Plant Cell.

[67] Xu K (2015). OsGRAS23, a rice GRAS transcription factor gene, is involved in drought stress response through regulating expression of stress-responsive genes. BMC Plant Biol.

[68] Achard P, Gong FS, Alioua M, Hedden P, Genschik P (2008). The Cold-Inducible CBF1 Factor-Dependent Signaling Pathway Modulates the Accumulation of the Growth-Repressing DELLA Proteins via Its Effect on Gibberellin Metabolism. Plant Cell.

[69] Jiang C, Fu X (2007). Phosphate starvation root architecture and anthocyanin accumulation responses are modulated by the gibberellin-DELLA signaling pathway in Arabidopsis. Plant Physiology.

[70] Punta M (2012). The Pfam protein families database. Nucleic Acids Res.

[71] Jin J, Zhang H, Kong L, Gao G, Luo J (2014). PlantTFDB 3.0: a portal for the functional and evolutionary study of plant transcription factors. Nucleic Acids Res.

[72] Jeanmougin F, Thompson JD, Gouy M, Higgins DG, Gibson TJ (1998). Multiple sequence alignment with Clustal X. Trends in Biochemical Sciences.

[73] Saitou H (1987). The neighbor-joining method: a new method for reconstructing phylogenestic trees. Mol. biol. evol.

[74] Tamura K (2011). MEGA5: molecular evolutionary genetics analysis using maximum likelihood, evolutionary distance, and maximum parsimony methods. Molecular Biology & Evolution.

[75] Franceschini A (2013). STRINGv9.1: protein-protein interaction networks, with increased coverage and integration. Nucleic Acids Res.

[76] Li N (2016). Effects of sunlight on gene expression and chemical composition of light-sensitive albino tea plant. Plant Growth Regulation.

[77] Wang YX, Liu ZW, Wu ZJ, Li H, Zhuang J (2016). Transcriptome-Wide Identification and Expression Analysis of the NAC Gene Family in Tea Plant [*Camellia sinensis* (L.) O. Kuntze]. Plos One.

[78] Wu ZJ, Tian C, Jiang Q, Li XH, Zhuang J (2016). Selection of suitable reference genes for qRT-PCR normalization during leaf development and hormonal stimuli in tea plant (*Camellia sinensis*). Sci Rep.

[79] Wu ZJ (2016). Transcriptome-wide identification of Camellia sinensis WRKY transcription factors in response to temperature stress. Molecular Genetics and Genomics.

[80] Pfaffl M (2013). A new mathematical model for relative quantification in real-time RT-PCR. Nucleic Acids Res. Nucleic Acids Res.

