# Supplementary material for: Genome-wide identification and expression analysis of GRAS family transcription factors in tea plant (Camellia sinensis)
Source: Sci Rep. 2018 Mar 2;8:3949. doi: 10.1038/s41598-018-22275-z (PMC5834537; doi:10.1038/s41598-018-22275-z)

**Genome‑wide identification and expression analysis of GRAS family transcription factors in tea plant (*Camellia sinensis*)**

Yong-Xin Wang, Zhi-Wei Liu, Zhi-Jun Wu, Hui Li, Wen-Li Wang, Xin-Cui, Jing Zhuang *

*Tea Science Research Institute, College of Horticulture, Nanjing Agricultural University, Nanjing 210095, China*

*Please address all correspondence to: J. Zhuang (zhuangjing@njau.edu.cn)

---------------

Dr. Jing Zhuang

Professor

Tea Science Research Institute,

College of Horticulture,

Nanjing Agricultural University,

Nanjing, 210095, China

Fax: 86 25 84395182

Email: [zhuangjing@njau.edu.cn](mailto:zhuangjing@njau.edu.cn)

**
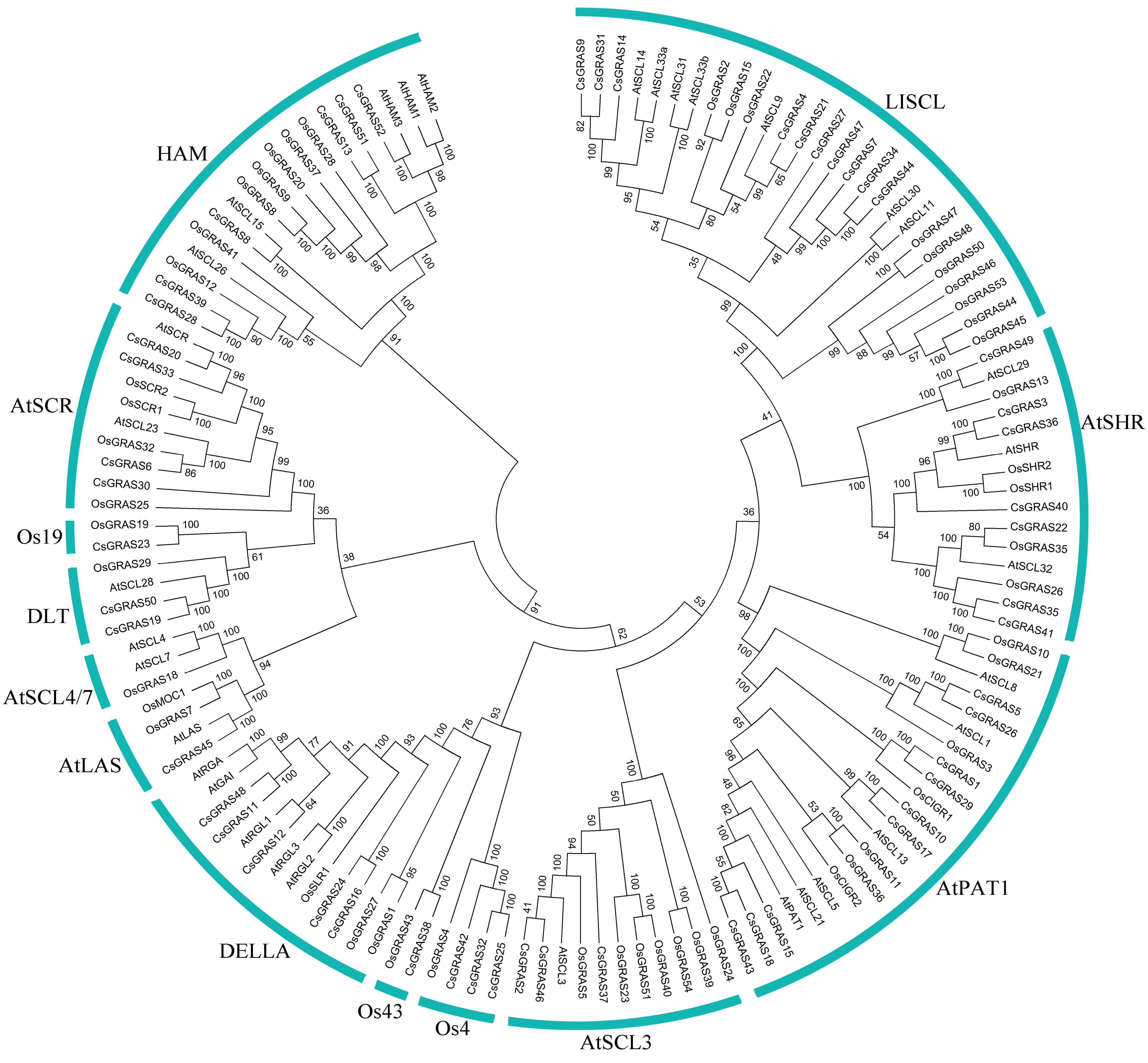
**

Fig S1. Phylogenetic analysis of GRAS proteins from tea plant, Arabidopsis, and rice. The full-length GRAS protein sequences were aligned using Clustal 1.83, and the phylogenetic tree was constructed using MEGA 5.05 by the minimum evolution (ME) method with 1000 bootstrap replicates.


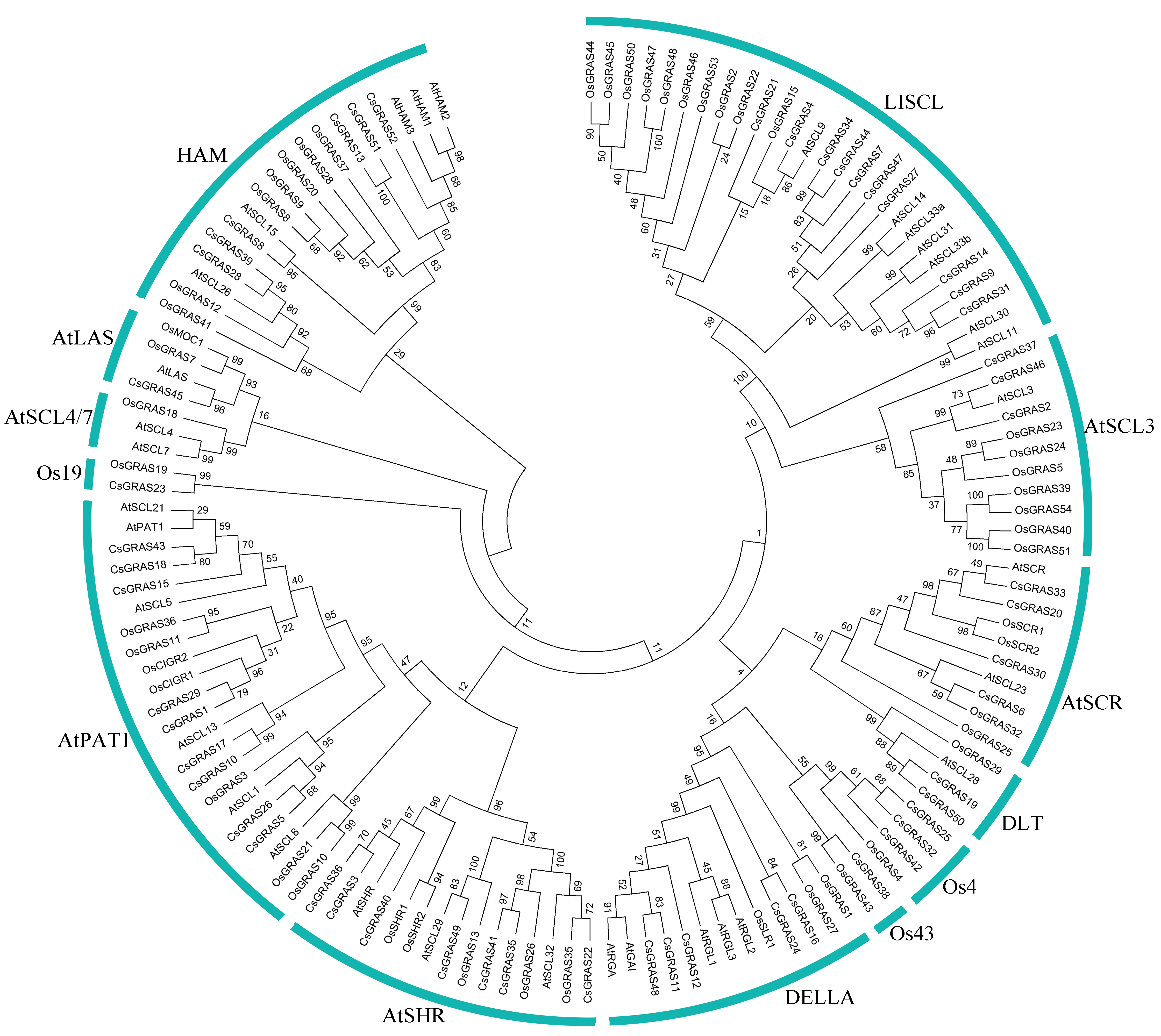


Fig S2. Phylogenetic analysis of GRAS proteins from tea plant, Arabidopsis, and rice. The full-length GRAS protein sequences were aligned using Clustal 1.83, and the phylogenetic tree was constructed using MEGA 5.05 by the maximum likelihood (ML) method with 1000 bootstrap replicates.


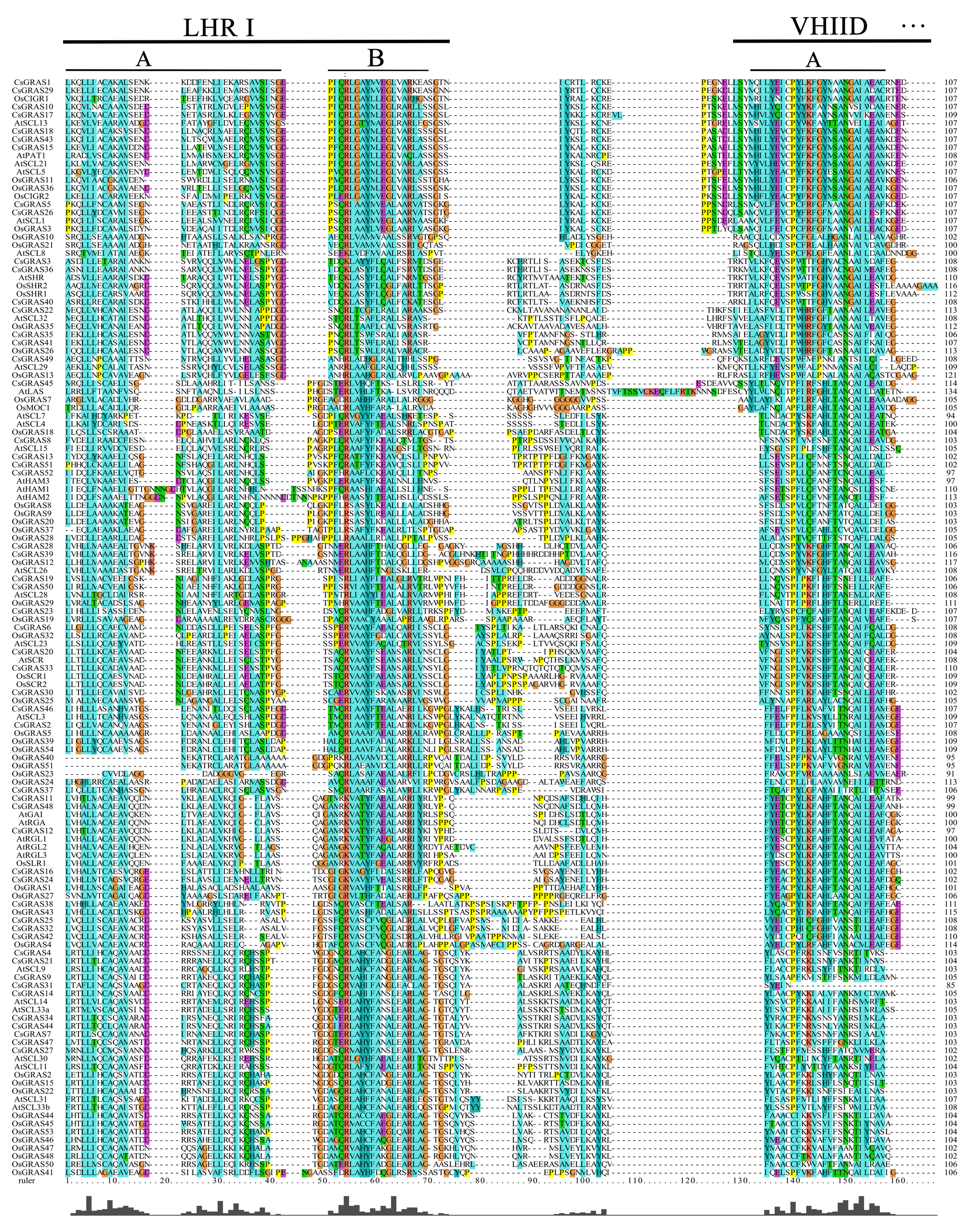


continue to next page

**
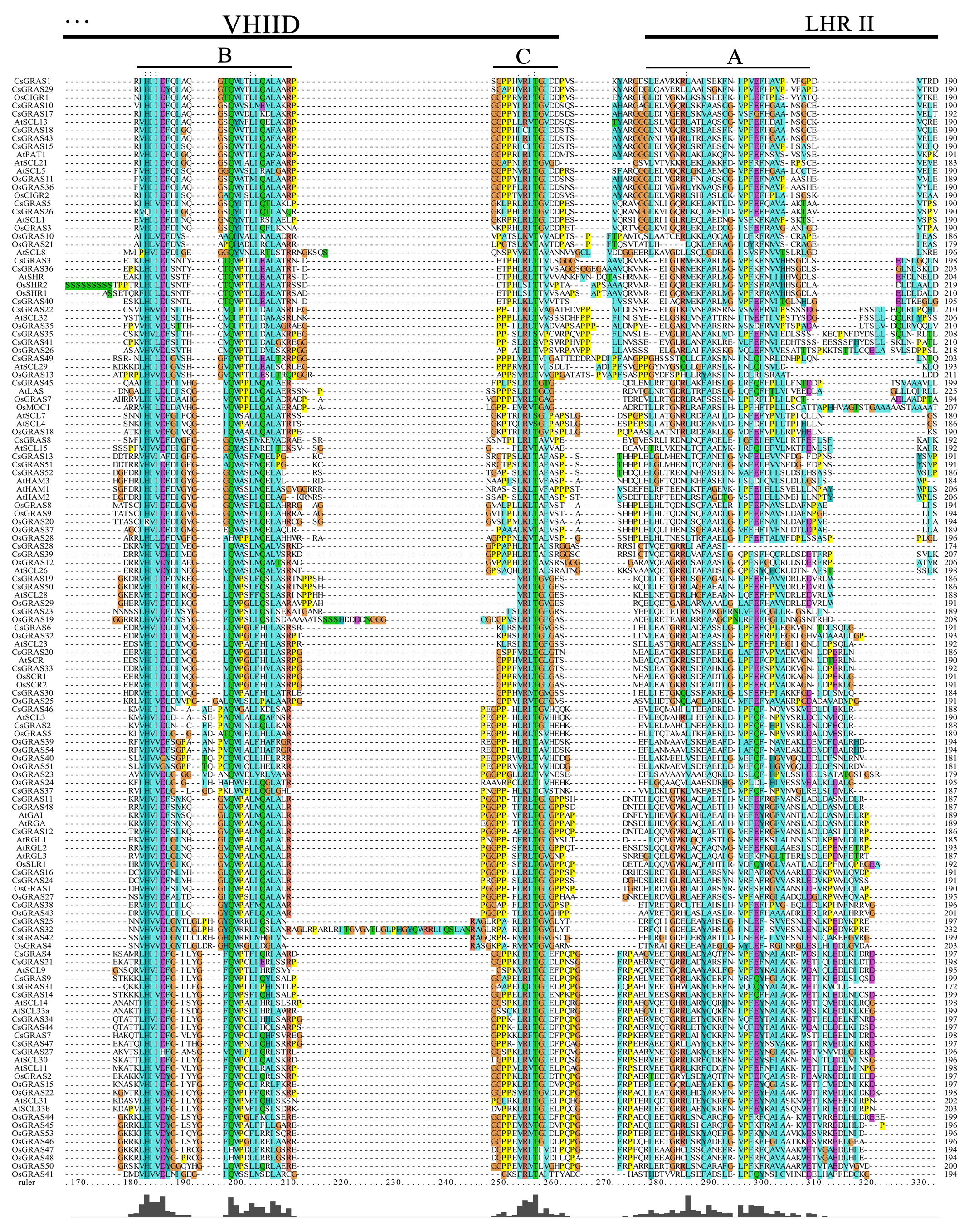
**

continue to next page


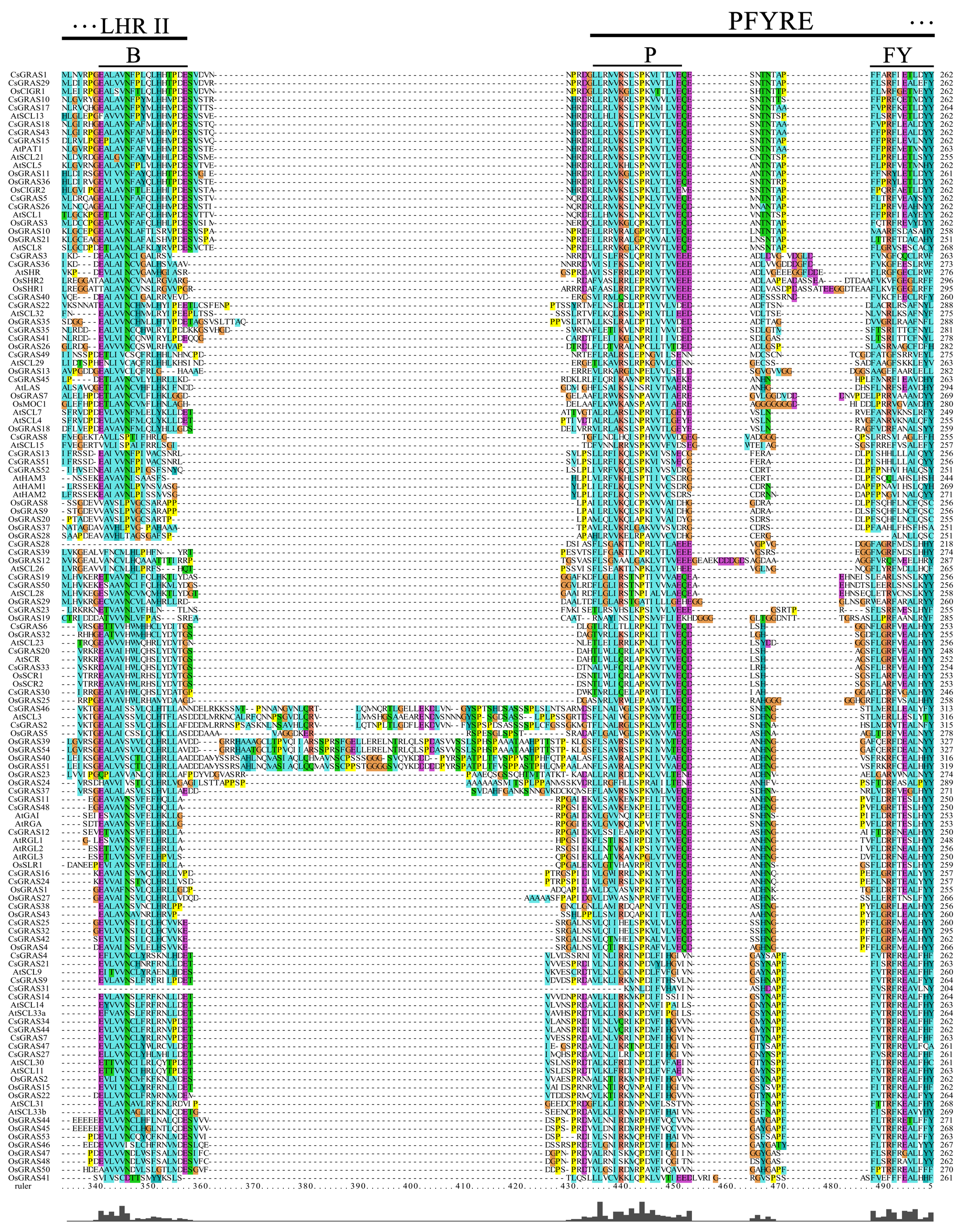


continue to next page

**
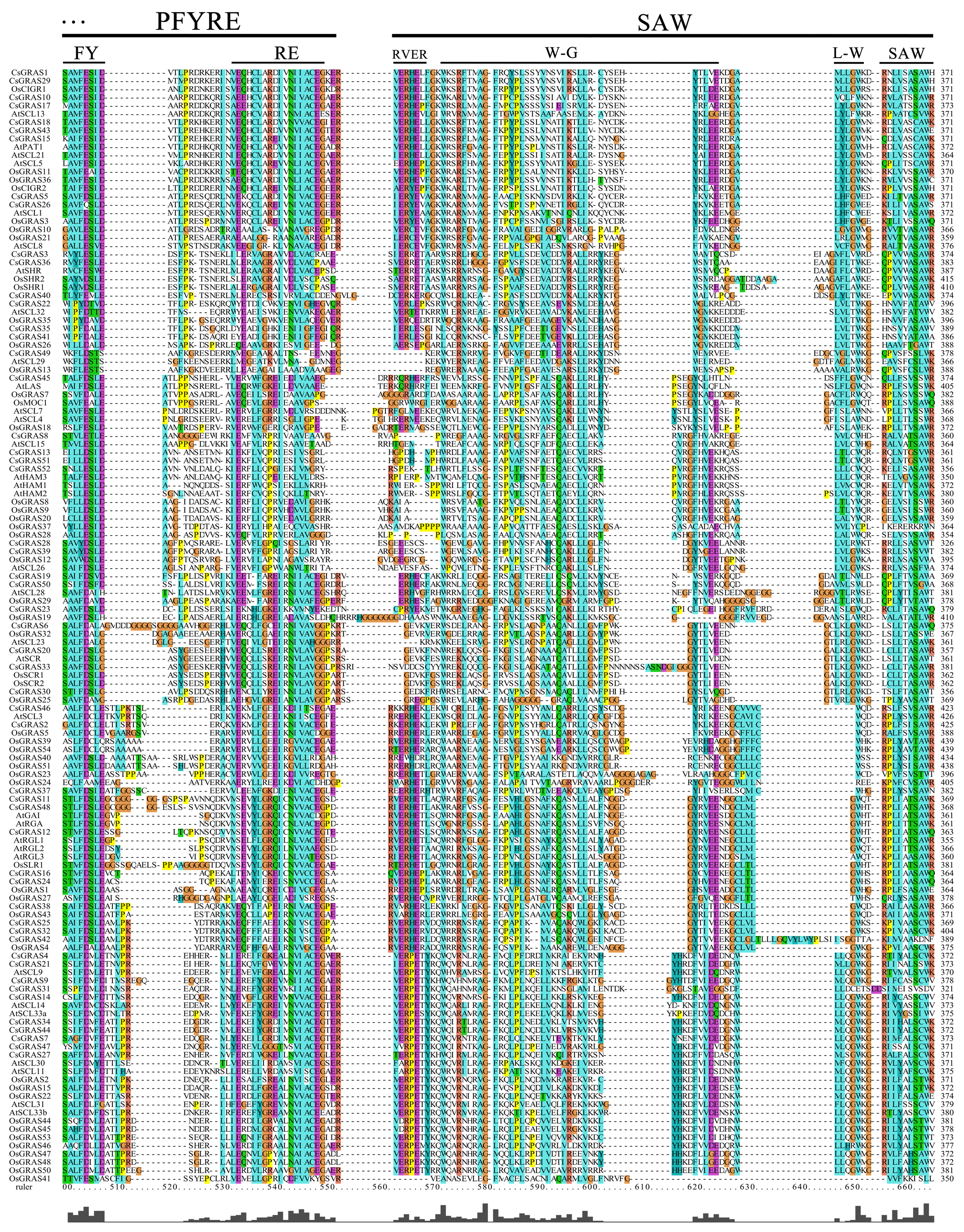
**

Fig S3. Multiple alignment of 135 GRAS proteins from tea plant, Arabidopsis, and rice. The conserved C-terminal GRAS domain is divided into five subdomains (LHRI, VHIID, LHRII, PFYRE, and SAW), which are indicated by lines above the sequences. LHR I and LHRII are composed of two repeat units (A and B); VHIID is further divided into three units (A, B, and C); PFYRE contains three distinct parts (P, FY, and RE); SAW is composed of four units (RVER, W-G, L-W, and SAW).

**
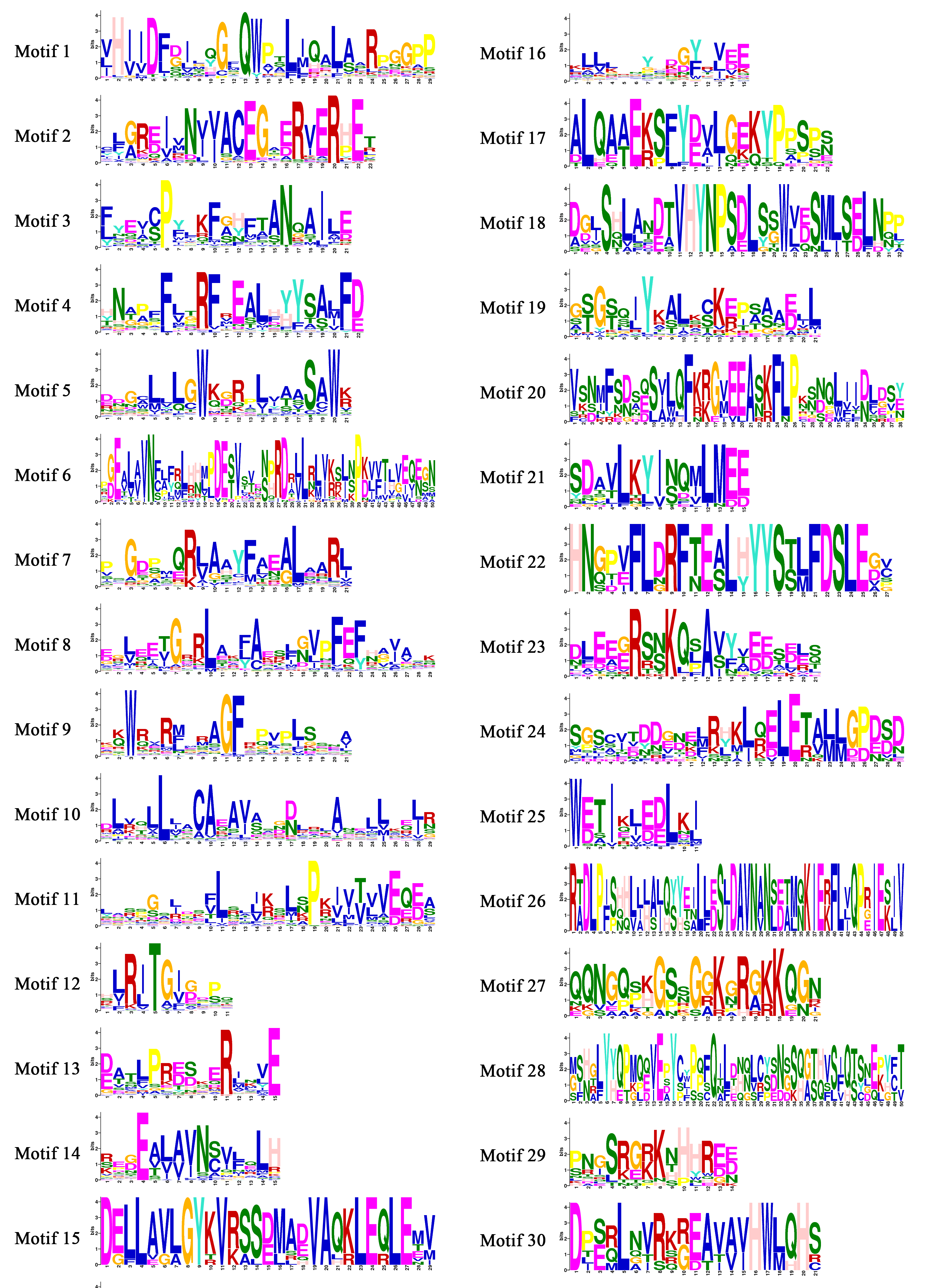
**

Fig S4. Sequence logos of conserved motifs identified in GRAS proteins from tea plant and Arabidopsis.


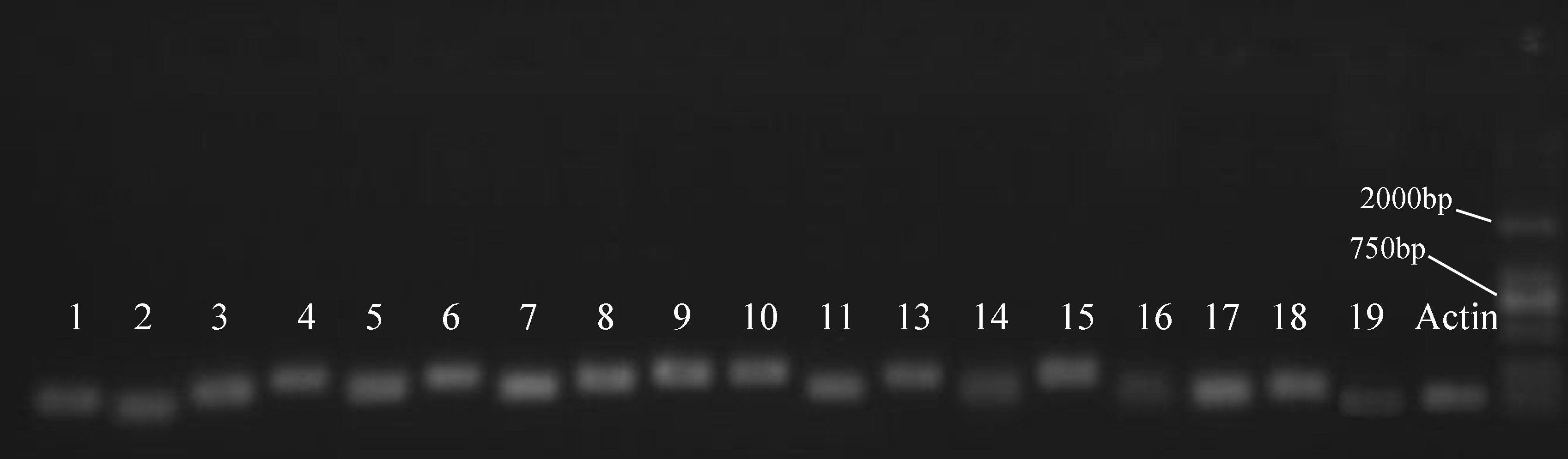


Fig S5. Electrophoresis results of all of the designed primers used for qRT-PCR.

Fig S6. Sequencing results of all of the *CsGRAS* squences used for qRT-PCR.

>CsGRAS1

ACTCTTCTATCCCACAGTTCCATCAATACCTGCTAGGCTGCTCGAGTCCTTGAAATTTGGTTTACATAACTCGCCTAACTCTCCTTTCTCCACTTACTTTGACTTTGAGACCCTTACCACATTGAGTAAA


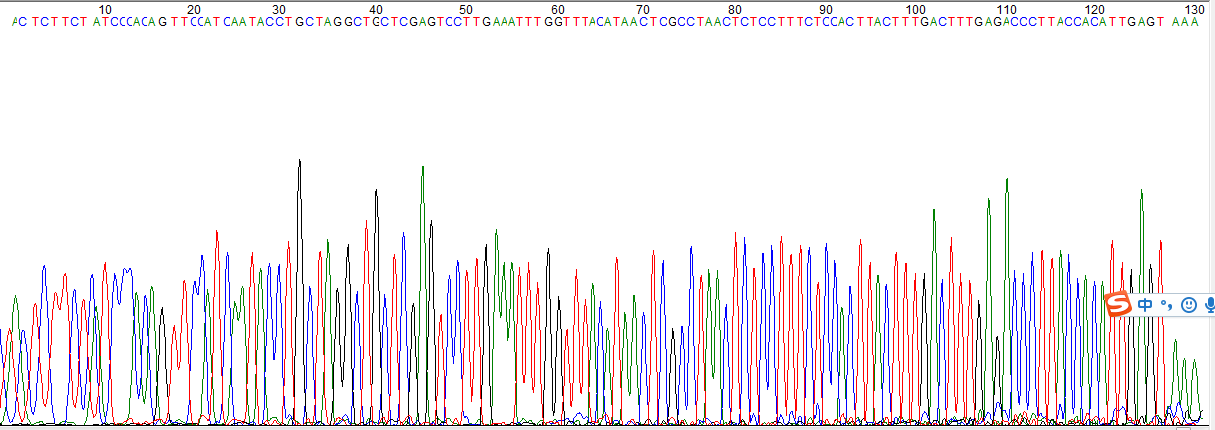


>CsGRAS2

TTTCTCCcTGATGACACTTTCACCTGGTATAGGTTCACCATTTCCGTGGA


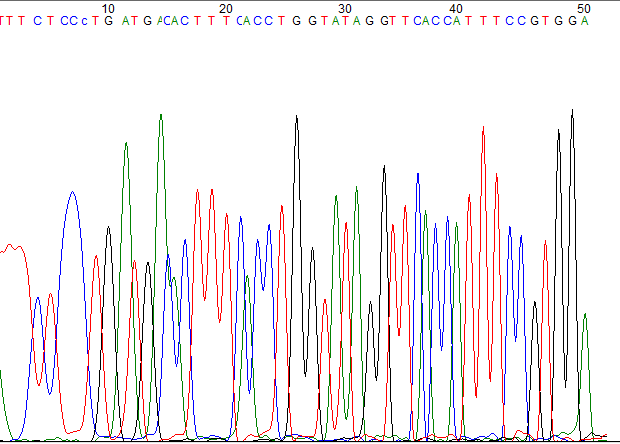


>CsGRAS3

CTAGACaACAAAACCACCATCATCAAGAAGAAGAAGAATGCTTCAACAATTTCATGGATGAAGATGGCTTCTCTTCTTCTTCTTCTAAGCACTACTACCCTTACCACCACCACCAACAACTCTCCA

‘
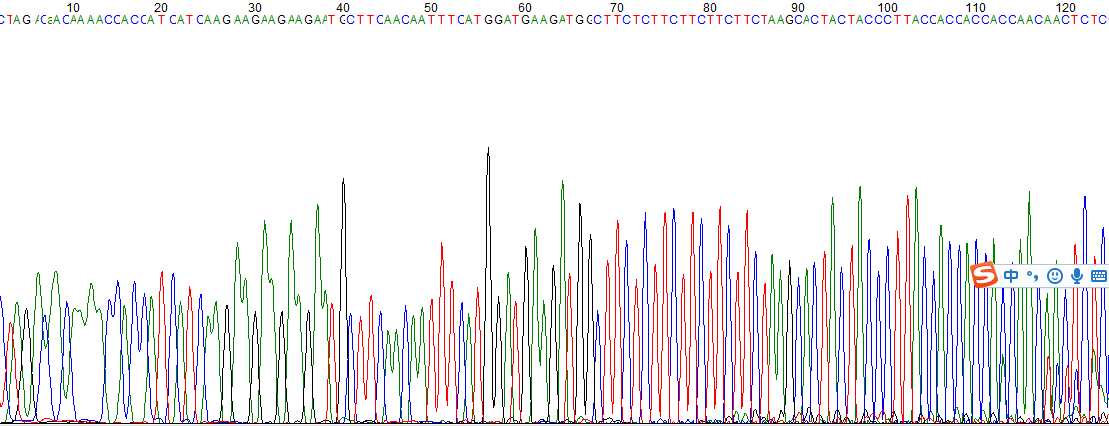


>CsGRAS4

ACTTCATCAAATAGTGTGAACAAAAATATTGATGGGTTCATGGATTCTCCAATAAGCCCTCTTCAGGTCACTGATTTATATAGTGGGAACCAATCTATT


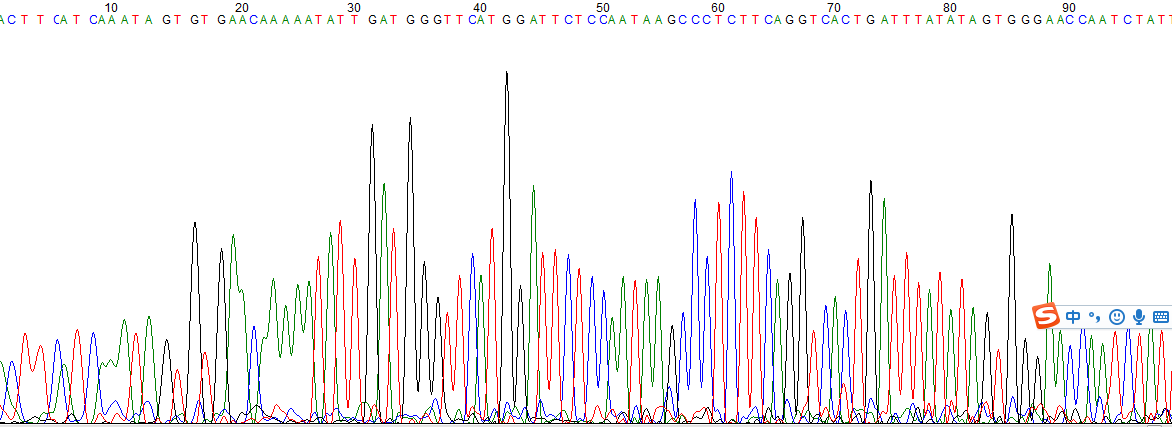


>CsGRAS5

ATTTATGCATTCATCTAACTCTGCTGACTCAGTGAATCGATTCCACCCACAAGGTGTCTCTTCTTACAATCATCGGATGGCTGGTTCGAGGCCAGTCATGACTGCACAAAACCCATTTAACTCTTTTGTGGGTATAAGGCATCAGGATGGGTATCA


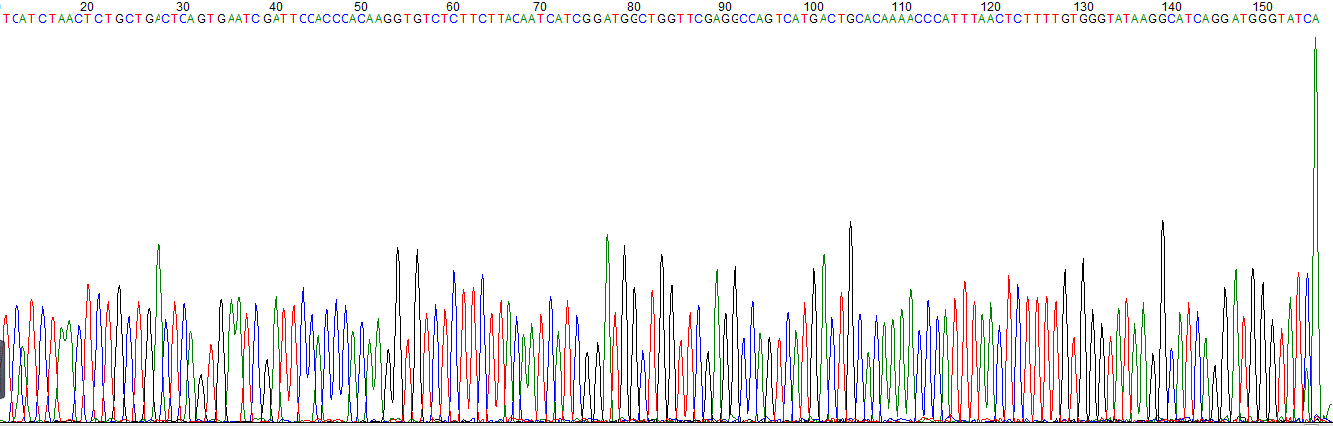


>CsGRAS6

CGaACCAAGCGCAGCCTCGACCTCGCCGCTGCCGGTGGCGAGTCCTCCTGTGAACAGGACGAGGATTCTTCAAGGAAGCGCCGGAATTACTCCGTCGACGAAGCCACAATTTCCGGCGAGAAGACCACCGGAGAACACGAGGAACGAGGCGAAGAAGCGGTGGTGATCGAAGGAGAGTCGTCAGGGTTGA


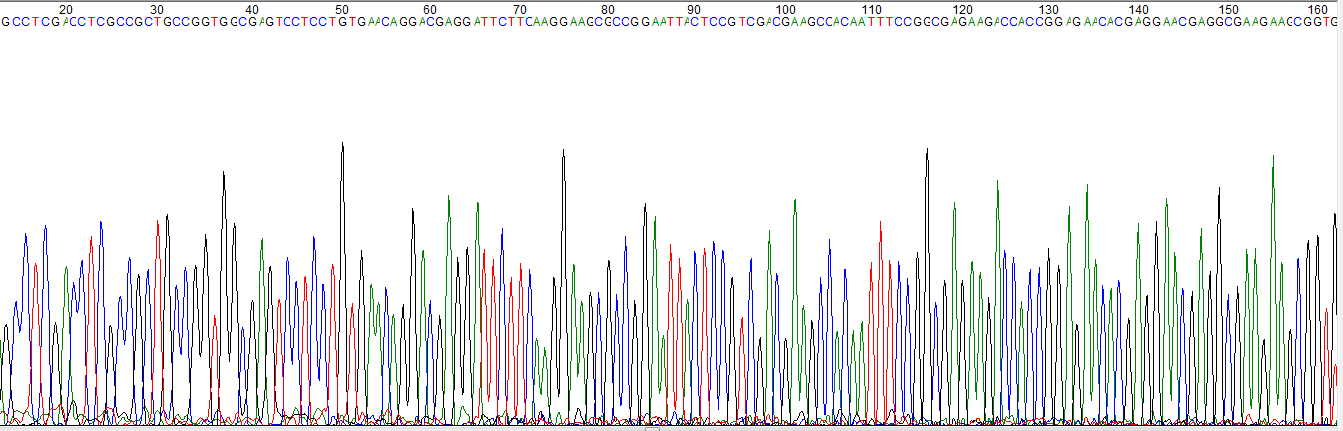


>CsGRAS7

ACGCACAATCGAGAAGACGCCGATTACACAGACGGTGCTGGCCGGAGTAACAAGCAATTGGCTAGTTTTGCTGAAGAGGCCGATGATCAATTGTACGATAAGGTGTTACTCTGTCCTCGCTTGAATCCTCATTTGA


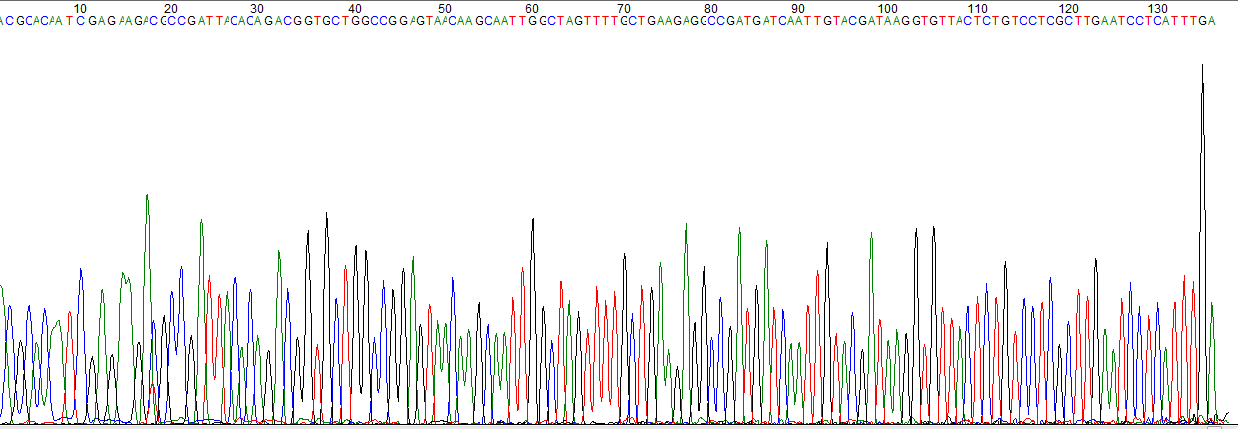


>CsGRAS8

ACAACAAAACAGTAGTGCTCGGAACACAAGGTTTAACAGCTCAACGGCCGATACCAACAACTTGTGTTACGAGCCTAAGTCGGTGCTAGACCTCCGCCGGAGCCAGAGCCCCATTAGCTCAGTCGCCGTGGCCGATAAGAAAGTCCCCAAGTCCGGTTTTTCGGATGCTTTGTTACAGTCGA


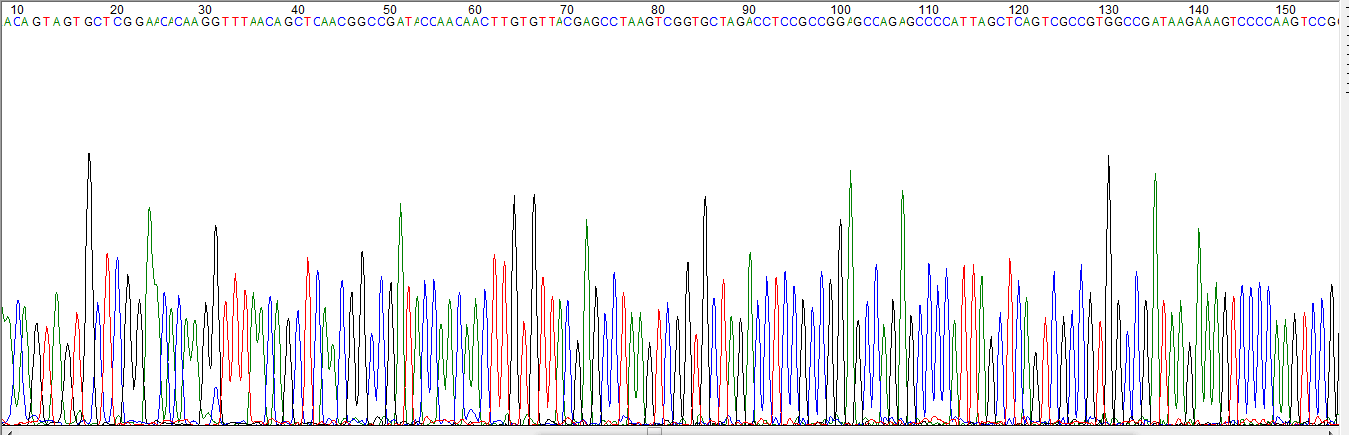


>CsGRAS9

CCTGCCTCCcCCAATCAACCATCCCCTCTTTACTTCAATCCAAATGTTGAAAGCCCTGATTTTTTATTTGGTGGTTCCAGTGAGCAGAGTGCTGGAACCAATACTAACGGTACCAATTCCATTGATTCTCAATGGATTGTTGACCCAGGAGAGCAAAAACAATCTGTACTAAGTTATCCTTCCGGATACCCTTTTGAGACCACTTTGAAACCTACTTCGCAGTTGT


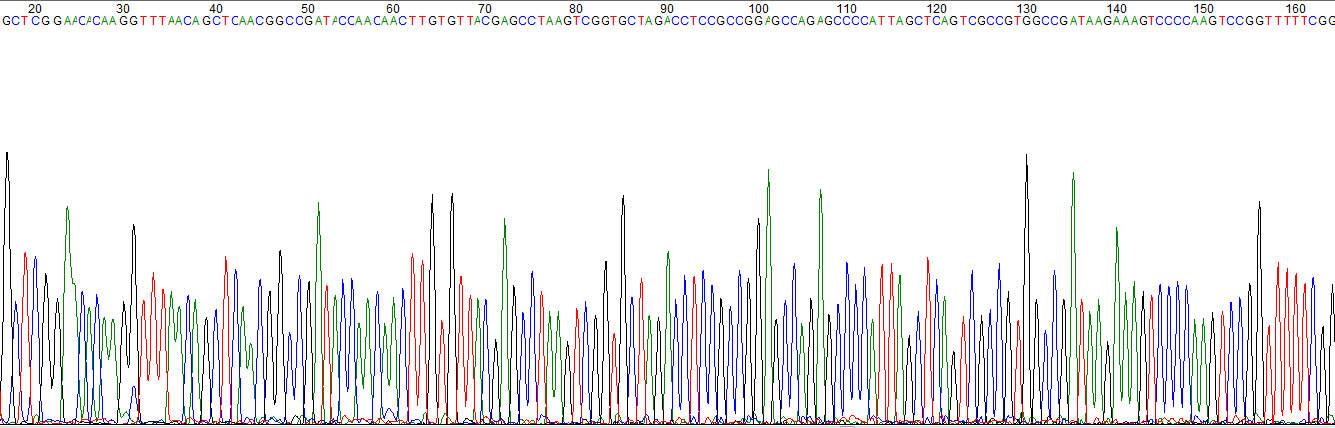


>CsGRAS10

ACCAGaACTATGCAGCAAGTCGAACCATACTGCTTCCCTTCTTTCCAAATTTTGCACAACAATTTGTGTTCTGATAATGACAAGCAAGCAACCCAAGTTTCCTTCCAATCTTGCAATGAACCACACTTTACTCTGGAATCTTCTCCAGCATCTGGTTATGTAGTTTATGATTCCCCTTCTGCTATGAGCTTCTCATCTAACAGGACTCCCATTTCGCCACTTGGTTCTCAGTCATACCTGTCGGAAAAG


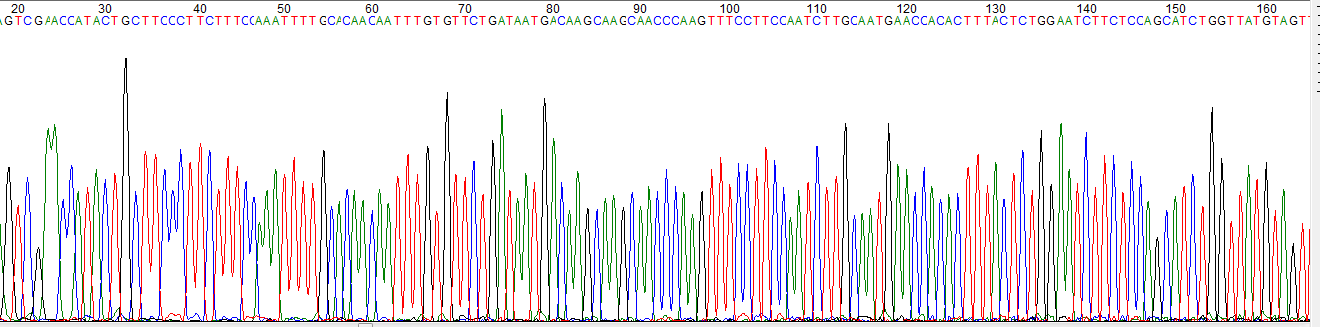


>CsGRAS11

CTTTTGCGGGGGCGGCGATGATGGCGTTGGGAGCTCCTCTGGCGGCGGCGGAAGTGGAAAACCCAAGACATGGGAAGAAGATGCCGGAGTGGATGAGCTCCTGGCCGTGTTGGGTTACAAGGTGAAGGCCTCCGACATGGCTGATGTTGCTCAGAAGCTGGAACAA


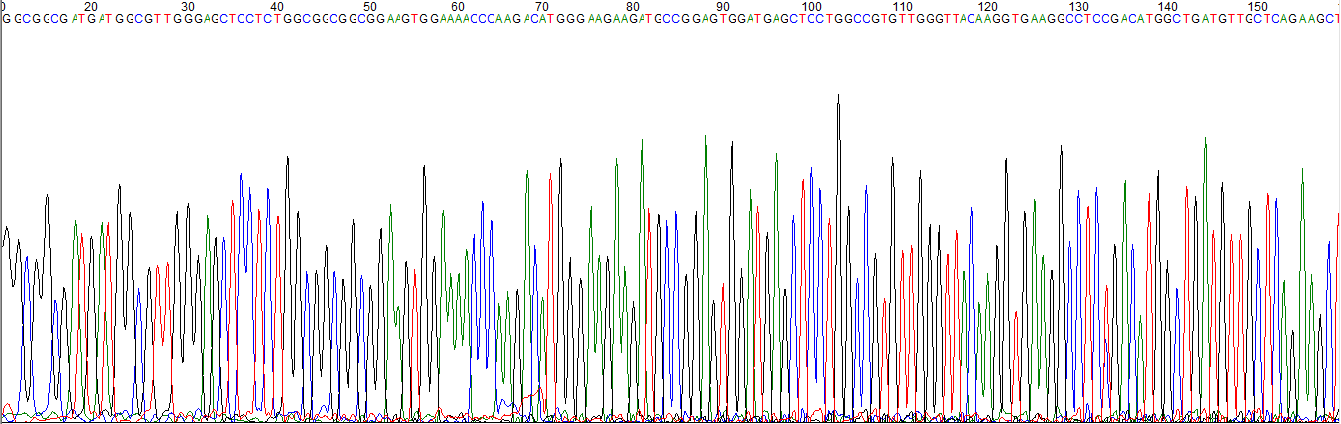


>CsGRAS13

AGCTTCAAATCTTGGGTTCAACAACAACAACACACAAATCCCCATTTTGGGTTCTTCAATCAACAATCTCTTTTCTAGTGATTTGATCTACCAGCTTGAAAACCCAGAGTCAAAGCCTCAGATTTTCAATCCACAAGTATTCATAAACCGACACCAACAACTTTCTCAGCTGTCTCAGACTCAAATTCAAACACAACCCTATCAACACCATCTTA


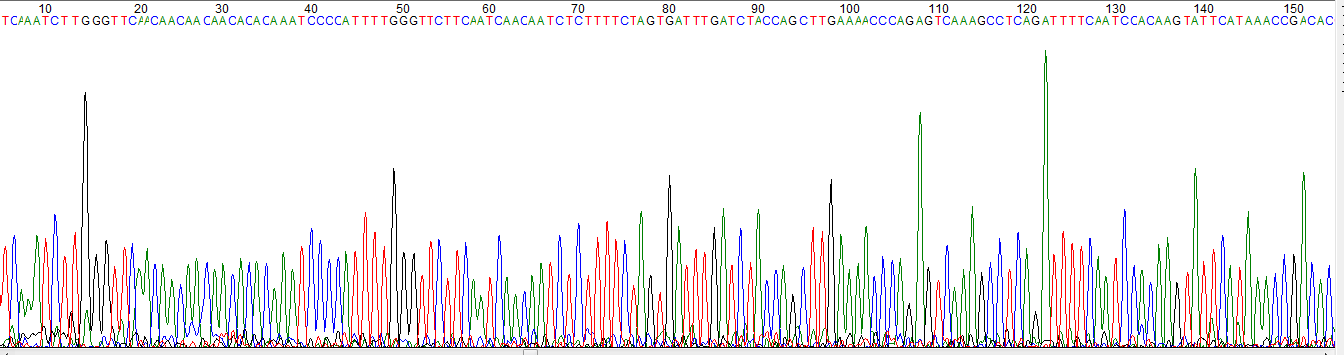


>CsGRAS14

ATccATGTGGTTGTTCATTCTGCTGCGAGCTCTTGGATGCTTCACTTTGCACTTCATCACCATAACAGCATGTATTCTGTCCTTTCCCTTCAGTAAAAAGTAAAACCTTATCAAACATTTCGGTTAACTCAGCCTCTTCCATAAAAACTGCTGACTGCTA


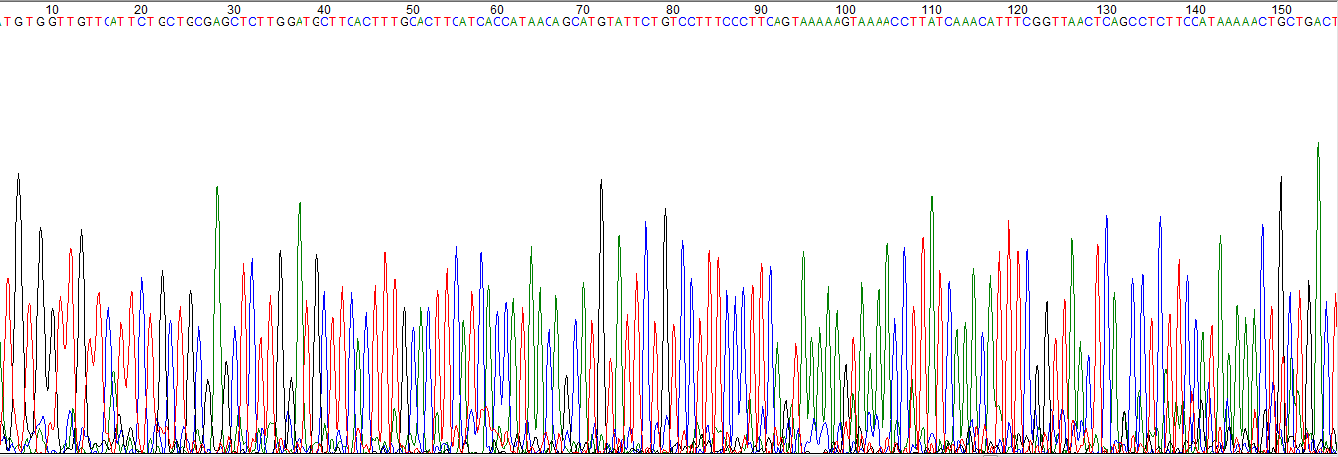


>CsGRAS15

TAGaACACGGGTTATGCTCCGATGATGGAAGCCAAGGGACCCATGTTTCTGTTCAGACGTCCGATCAACTTGGCACTGTTGACTCATCCACAGCAACTGGTGGTTACCCTTTTCAAAATTCTCCGTCTACCGTGAGTTTCTCTCCGCATGGAAGCCCCACCTCTCAGCCTGATTCTCATTCATATGCATCTGACATGCATCATTCACCAGACAATACTATCAGCTCGGCCATAAGTGGGATTTgCTTGACGGACA


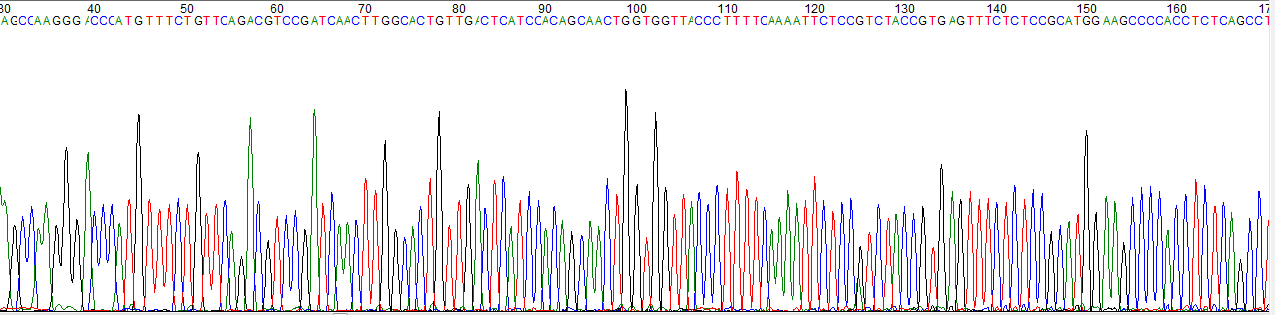


>CsGRAS16

CCATGACCCGGTGTTATTAACCGTCGGATCTAATGCGGGTCCCATA


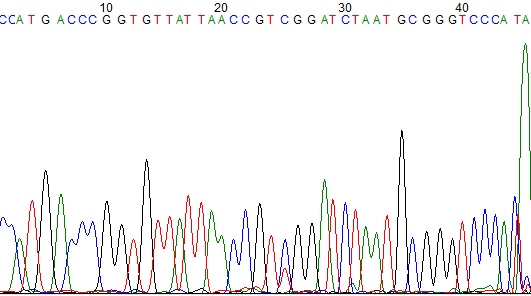


>CsGRAS17

CGTACTTCaCACTGGAATCGTCTCCAGCAAACGGTTACATTGTCTATGATTCCCCTCCTGCTGTGAGCATCTCTTCTAACCGGACTCCCTTTTCACCACAAGGTTCTCAGTCA


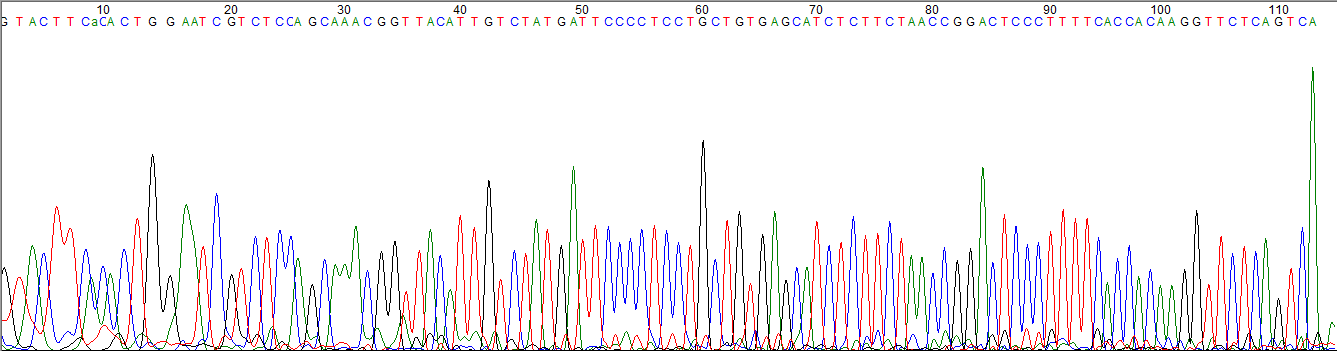


>CsGRAS18

TCTCAGTTTCTCACCCAATGGAAGTCCCATATCTCAGCAAGAGTGTCATTCATATGGGTCCTGTGTAACTGATGATTTTATTGAATTCAGACACAAGCTGCGTGAACTT


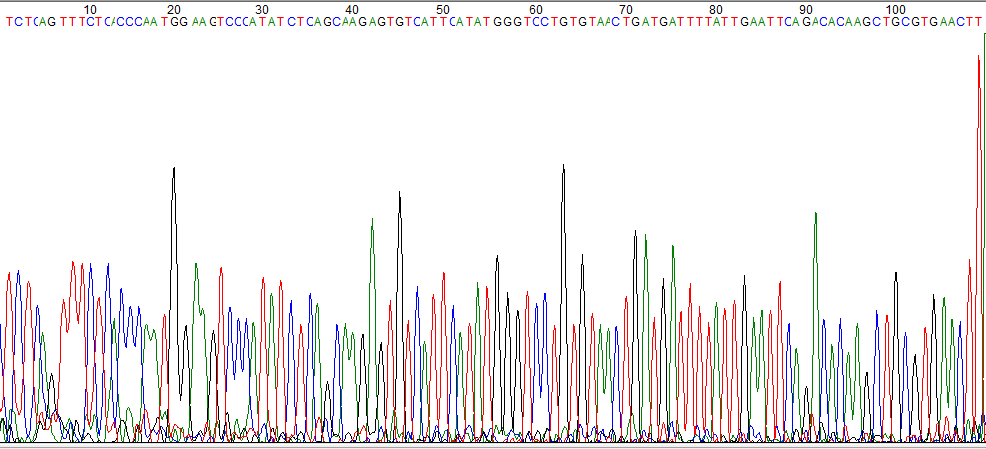


>CsGRAS19

ATGATTCTCATTCTCCAAAAGGCGGTGCAGACGAAGAATTGAGGAGTTTAGGCCATCTGGGTAGTGGGAATTTTTGGTTTCAGCCAAGTTTTGAGA


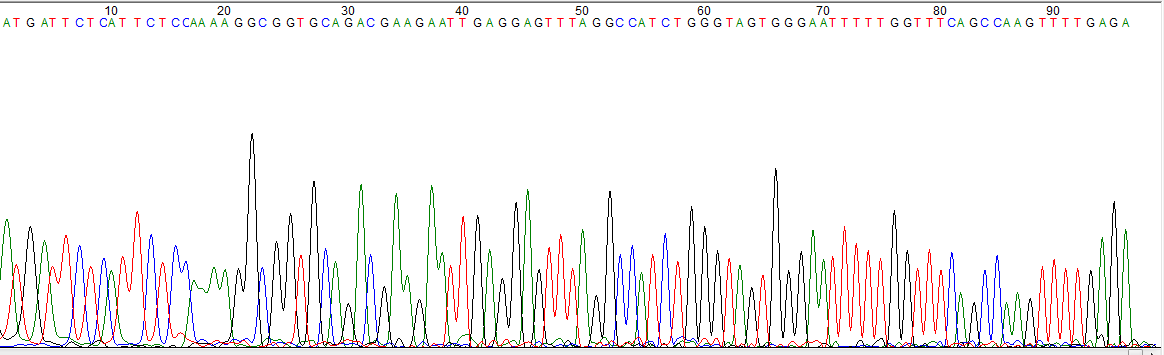

Supplement: Supplementary file 1 — Supplementary Figures [file 41598_2018_22275_MOESM1_ESM.doc]
